# Extracellular vesicle‐packaged ILK from mesothelial cells promotes fibroblast activation in peritoneal fibrosis

**DOI:** 10.1002/jev2.12334

**Published:** 2023-06-26

**Authors:** Qiang Huang, Yuxiang Sun, Long Peng, Juan Sun, Zixin Sha, Hongchun Lin, Yongjie Li, Canming Li, Huiqun Li, Hongli Shang, Yanxu Chen, Xianrui Dou, Zhaoyong Hu, Zengchun Ye, Hui Peng

**Affiliations:** ^1^ Nephrology Division, Department of Medicine the Third Affiliated Hospital, Sun Yat‐sen University Guangzhou China; ^2^ Division of Cardiovascular Medicine, Department of Medicine the Third Affiliated Hospital, Sun Yat‐sen University Guangzhou China; ^3^ Department of Biological Sciences Carnegie Mellon University Pittsburg Pennsylvania USA; ^4^ Organ Transplant Center the First Affiliated Hospital, Sun Yat‐sen University Guangzhou China; ^5^ Department of Nephrology Shunde Hospital, Southern Medical University (The First People's Hospital of Shunde) Foshan China; ^6^ Nephrology Division, Department of Medicine Baylor College of Medicine Houston Texas USA; ^7^ NHC Key Laboratory of Clinical Nephrology (Sun Yat‐sen University) and Guangdong Provincial Key Laboratory of Nephrology Guangzhou China

**Keywords:** cell‐cell communication, extracellular vesicles, ILK, peritoneal dialysis, peritoneal fibrosis, peritoneum

## Abstract

Progressive peritoneal fibrosis and the loss of peritoneal function often emerged in patients undergoing long‐term peritoneal dialysis (PD), resulting in PD therapy failure. Varieties of cell‐cell communications among peritoneal cells play a significant role in peritoneal fibrogenesis. Extracellular vesicles (EVs) have been confirmed to involve in intercellular communication by transmitting proteins, nucleic acids or lipids. However, their roles and functional mechanisms in peritoneal fibrosis remain to be determined. Using integrative analysis of EV proteomics and single‐cell RNA sequencing, we characterized the EVs isolated from PD patient's effluent and revealed that mesothelial cells are the main source of EVs in PD effluent. We demonstrated that transforming growth factor‐β1 (TGF‐β1) can substitute for PD fluid to stimulate mesothelial cells releasing EVs, which in turn promoted fibroblast activation and peritoneal fibrogenesis. Blockade of EVs secretion by GW4869 or Rab27a knockdown markedly suppressed PD‐induced fibroblast activation and peritoneal fibrosis. Mechanistically, injured mesothelial cells produced EVs containing high level of integrin‐linked kinase (ILK), which was delivered to fibroblast and activated them via p38 MAPK signalling pathway. Clinically, the expression of ILK was up‐regulated in fibrotic peritoneum of patients undergoing long‐term PD. The percentage of ILK positive EVs in PD effluent correlated with peritoneal dysfunction and the degree of peritoneal damage. Our study highlights that peritoneal EVs mediate communications between mesothelial cells and fibroblasts to initiate peritoneal fibrogenesis. Targeting EVs or ILK could provide a novel therapeutic strategy to combat peritoneal fibrosis.

## 1 INTRODUCTION

Peritoneal dialysis (PD) is a common renal replacement therapy option for approximately 3.8 million patients with end‐stage renal disease (ESRD) worldwide (Teitelbaum, 2021). However, the loss of peritoneal function and ultrafiltration failure caused by peritoneal fibrosis limit the application of PD (Krediet, 2018). Fibrosis is a reparative response to tissue injury, which often results in development of fibrous connective tissue (Edeling et al., 2016). During PD, injured peritoneal mesothelial cells trigger a cascade of cellular responses via various intercellular communications with macrophages, endothelial cells, and fibroblasts (Helmke et al., 2019; Shi et al., 2022; Witowski et al., 2015). Among them, the activation of fibroblasts is a key step in the progression of peritoneal fibrosis (Chen et al., 2014). However, how injured mesothelial cells transmit signals to activate fibroblasts remains elusive.

Extracellular vesicles (EVs) have emerged as important mediators of intercellular communication (Lv et al., 2020). Based on the biochemical and biophysical characteristics, EVs are classified into two main EV subpopulations including exosomes and ectosomes (Niel et al., 2022). Carrying a variety of molecular cargo, such as proteins, DNAs, RNAs and metabolites, EVs are ubiquitously produced by most cell types and mediate cell‐cell crosstalk by modulating the behaviour of recipient cells via receptor‐ligand interaction, direct membrane fusion or endocytosis (Lei et al., 2021; Ruivo et al., 2022). In addition to this functional role, EVs can be excreted into various body fluids, such as blood and urine, which can be used as novel non‐invasive biomarkers to indicate the pathophysiology (Merchant et al., 2017; Thietart & Rautou, 2020).

Mounting evidence has revealed that peritoneal cells respond to the bioincompatible dialysate exposure by releasing various EVs into PD effluent (Yu et al., 2018). These EVs in PD effluent are identified as disease‐related biomarkers for patients undergoing PD. For example, exosomes containing aquaporin 1 from PD effluent can properly reflect dialysis efficiency and the status of peritoneum (Corciulo et al., 2019). Similarly, EVs‐packaged glycoprotein 96 from PD effluent is also a potential biomarker of the peritoneal solute transport rate (Fang et al., 2022). Although these studies reflected the diagnostic value of EVs during PD, the information of whether and how EVs make use of their molecular cargos to influence the development of peritoneal fibrosis is still lacking.

In this study, we isolated EVs from peritoneal lavage fluid (PLF) and PD effluent. Using combined analysis of EV proteomics and peritoneal single‐cell RNA sequencing (scRNA‐seq), we identified mesothelial cells as the main source of PD effluent‐derived EVs and confirmed that long‐term PD stimulated the secretion of EVs in mesothelial cells. We also uncovered that EVs mediated the communication between mesothelial cells and fibroblasts, and demonstrated that mesothelial EVs transmitted their molecular cargo to activate fibroblasts. Inhibition of EVs secretion prevented the development of peritoneal fibrosis in a mouse model of peritoneal dialysis.

## 2 MATERIALS AND METHODS

### 2.1 Study design

The aim of the study was to investigate the role of mesothelial EVs in peritoneal fibrogenesis and identify potential therapeutic targets. Firstly, integrative analyses of EV proteomic data and scRNA‐seq data of normal peritoneal tissue were used to determine the main source of EVs in PD effluent. Secondly, primary cell culture and animal studies were performed to investigate the effects of mesothelial EV‐packaged ILK on fibroblast activation. Thirdly, relevance of ILK in clinical PD was evaluated. All samples were at least three independent biological replicates. More details are provided in each figure legend.

### 2.2 Human sample collection

All human peritoneum, PLF and PD effluent were obtained from inguinal hernia patients with normal renal function and patients undergoing PD at The Third Affiliated Hospital of Sun Yat‐sen University. The use of human tissue for our study was approved by the Ethics Committee of the Third Affiliated Hospital of Sun Yat‐sen University ([2022]02‐268‐01). Written consents were obtained from the patients. Total 18 samples of PLF, short‐term PD effluent (SPD, patients undergoing PD less than 1 year) and long‐term PD effluent (LPD, patients undergoing PD more than 4 years) were collected to isolate EVs by ultracentrifugation. The EV samples were performed with 4D label‐free proteomics analysis. A total of 64 samples of PLF, SPD and LPD effluent were used to evaluate the proportion of ILK^+^ EVs by flow cytometry and obtain the mesothelial cells. Human omentums from 12 volunteers were used to isolate primary mesothelial cells and fibroblasts.

### 2.3 EV isolation

EV isolation was performed for PLF and PD effluent samples. 400 mL of fresh fluid was centrifuged at 4°C at 300 × *g* for 5 min and 2000 × *g* for 20 min, and 10,000 × *g* for 30 min. The supernatant was filtered using a 0.22 μm filter (Merck Millipore, SLGP033RB). Next, samples were ultracentrifuged at 100,000 × *g* for 90 min at 4°C in a SW28 Ti rotor (Beckman Coulter). The precipitate was resuspended in phosphate buffered saline (PBS) and ultracentrifuged at 100,000 × *g* for another 90 min. Finally, the precipitate was resuspended in 100 μL of PBS.

Mesothelial EVs were obtained from cell conditioned medium. Mesothelial cells were treated with 10 ng/mL TGF‐β1 or not for 24 h, washed with PBS and then cultured in serum‐free medium for 48 h. Conditioned media was collected to isolate EVs by ultracentrifugation as above mentioned. Isolated mesothelial EVs were added into fibroblasts at a concentration of 30 μg/mL.

### 2.4 Transmission electron microscopy (TEM)

EV samples were dried on a copper grid for 5 min. After fixation with 2.5% glutaraldehyde for 20 min, the samples were stained with uranyl acetate and observed using a transmission electron microscope (Hitachi, HT‐7700) at 100 kV.

### 2.5 Nanoparticle tracking analysis (NTA)

The concentration and size distribution profiles of EVs were obtained from NanoSight NS300 instrument (Malvern, Worcestershire, UK). EVs were pre‐diluted to 1 mL, to achieve a concentration within the 10^7^–10^8^ range. Diluted Samples were injected into the sample‐carrier cell slowly and the particles were automatically tracked and measured using Brownian motion and diffusion coefficients. The parameters were set at 25°C ± 0.5°C with a camera level at 14–15. The size (nm) and concentration (particles/mL) of EVs were calculated by integrating the data from three records with a detection threshold 4/5.

### 2.6 4D label‐free proteomics analysis

The primary experimental processes for proteomics contain protein extraction, quantification, quality inspection, trypsin digestion, LC–MS/MS and data analysis. Available proteins from the LC‐MS/MS data were specified by the following criteria: the protein was detectable in ≥3 samples of any given group or ≥5 samples in the total. Filtered proteins of PD effluent‐derived EVs were used as input datasets. Subsequently, datasets were processed with FactoMineR R package (version 2.4) to scaled and summarized by Principal Component Analysis. Visualization of the result was generated using functions from ggplot2 R package (version 3.3.3). Differential proteins among the groups were assessed with the edgeR R package (version 3.28.1). Benjamin & Hochberg false discovery rate (FDR) method was used as a *P* value adjustment. Adjusted *P* < 0.05 was considered statistically significant. Gene Ontology analysis was done by the clusterProfiler R package (version 3.14.3). Gene Set Variation Analysis was performed using the clusterProfiler function gsva, which based on the hallmark gene sets from the Molecular Signatures Database (MSigDB).

### 2.7 Single cell RNA sequencing analysis

scRNA‐seq datasets of normal human peritoneum and peritoneal cells from PD effluent were prepared from our prior study GSE130888 (Si et al., 2019). Seurat R package (version 3.2.3) was used for quality control and normalized steps. Batch effects were corrected using the Harmony R package (version 0.1.0). Subsequently, datasets were clustered by the Seurat FindNeighbors and FindClusters functions. Then, Uniform Manifold Approximation and Projection (UMAP) was used to perform dimensional reduction and canonical cell‐type markers were used for cluster annotation.

### 2.8 Integrative analysis of scRNA‐seq data and LC–MS/MS data

To evaluate the proportion of EVs derived from different cell types, cell type deconvolution of proteome data was performed using Multi‐subject Single Cell deconvolution (MuSiC) R package. Followed by the MuSiC workflow, normalized scRNA expression data of human peritoneum was used to train cell type composition for individual cell types.

In order to evaluate the EVs secretion ability of single cell of different cell types, we choose all protein in PD effluent‐derived EV proteome as the gene set of EVs secretion and clusterProfiler package was apply to perform single sample Gene Set Enrichment Analysis to calculate the enrichment score of each single cell in our scRNA‐seq datasets.

### 2.9 Primary culture of human peritoneal mesothelial cells and fibroblasts

To obtain human peritoneal mesothelial cells, pieces of 3 × 3 cm human omentum were rinsed and cut up, followed by digestion at 37°C for 20 min with gentle stirring using trypsin. After termination of the digestion with 10% foetal bovine serum (FBS, Gibco, 10270106), the suspension was filtered with a 70 μm cell strainer (Falcon, 352350) and centrifuged at 1200 rpm for 5 min. Subsequently, the cell precipitation was washed and isolated pure mesothelial cells by UPK3B antibody (Absci, AB35546) and flow cytometry. Isolated mesothelial cells were cultured in Earle's M199 medium (Gibco, C11150500BT) supplemented with 20% FBS, 100 U/mL penicillin and 100 μg/mL streptomycin (PS, Gibco, 15140122) and 1% Insulin‐Transferrin‐Selenium (ThermoFisher, 41400‐045).

Peritoneal fibroblasts were isolated from human omentum after removing mesothelial cells as described previously (Beavis et al., 1997). Tissues were minced and digested in trypsin at 37°C for 40 min. After termination of the digestion and filtration, the suspension was centrifuged and cells were cultured in DMEM medium (Gibco, C11995500BT) containing 20% FBS and 1% PS.

### 2.10 Cell culture and treatment

Human mesothelial cell line MeT5A and fibroblast cell line HFF‐1 were purchased from ATCC. MeT5A cells were cultured in Earle's M199 medium supplemented with 10% FBS, 1% PS. HFF‐1 cells were cultured in DMEM medium containing 10% FBS and 1% PS. All cell lines were maintained in a 5% CO_2_ and 37°C humidified incubator. In a 12‐well transwell system, mesothelial cells (3×10^5^) were added to the upper chamber and treated with 10 ng/mL TGF‐β1 for 24 h or pretreated with 20 μM GW4869, while fibroblasts were added to the lower chamber, the pore size was 0.4 μm. After 3 days of co‐culture, mesothelial cells were withdrawn and fibroblasts were collected.

### 2.11 Western blot

Protein extraction of cells and peritoneal tissues were prepared using radio‐immunoprecipitation assay (RIPA) lysis buffer (Beyotime, P0013B) containing protease and phosphatase inhibitor (Beyotime, P1045) for 10 min on ice. Samples were centrifuged at 13,000 × *g* for 10 min to collect supernatant. After that, protein concentration was measured with the bicinchoninic acid (BCA, Beyotime, P0010) kit and protein was denatured in loading buffer at 95°C for 5 min. Next, 10 μg of the protein was separated on sodium dodecyl sulphate polyacrylamide gel electrophoresis (SDS‐PAGE) and then transferred to a polyvinylidene difluoride (PVDF) membrane (Roche, 3010040001). Then, membranes were blocked by 5% bovine serum albumin (BSA) followed by incubation in primary antibodies at 4°C overnight. The next day, membranes were incubated with HRP‐conjugated rabbit (Boster, BA1054, 1:5000) or mouse (Boster, BA1050, 1:5000) secondary antibodies for 1 h. Subsequently, the bands were visualized by enhanced chemiluminescence (Elabscience, E‐BC‐R3470).

EV samples were also denatured in loading buffer at 95°C for 5 min and 10 μg of EV protein was loaded and separated as mentioned above.

The primary antibodies used for western blot were anti‐CD9 (CST, 13403S, 1:1000), anti‐CD63 (Proteintech, 25682‐1‐AP, 1:400), anti‐CD81 (CST, 56039S, 1:1000), anti‐CD81(CST, 10037S, 1:1000), anti‐TSG101 (Proteintech, 28283‐1‐AP, 1:2000), anti‐Calnexin (Proteintech, 10427‐2‐AP, 1:1000), anti‐Fibronectin (Abcam, ab2413, 1:3000), anti‐COL1A1 (CST, 72026S, 1:1000), anti‐α‐SMA (Sigma‐Aldrich, A5228, 1:2000), anti‐FAP (Abcam, ab207178, 1:2000), anti‐PDGFRB (CST, 3169S, 1:1000), anti‐ILK (Abcam, ab76468, 1:3000), anti‐β‐actin (CST, 8457S, 1:2000), anti‐GAPDH (CST, 5174S, 1:2000), anti‐β‐Tubulin (CST, 2146S, 1:2000).

### 2.12 Quantitative reverse transcription‐PCR

Total RNA was extracted with the TRIzol reagent (Invitrogen, 15596018), followed by reverse transcription using the PrimeScript™ RT reagent kit (Takara, RR047A). After that, quantitative real‐time PCR was performed utilizing a Roche LightCycler® 480 quantitative real‐time PCR system according to the standard protocol. The Ct values were normalized to β‐actin and the relative gene expression was calculated using the −ΔΔCt method. Briefly, Ct value of target gene of each sample minus the mean Ct value of the reference molecule in the same sample to the ΔCt value. The mean ΔCt value of target gene in control group was calculated and the ΔCt value of target gene of each samples minus the mean ΔCt value of target gene in control group to the ΔΔCt value. The relative gene expression was calculated by 2^‐ΔΔCt.

### 2.13 EVs fluorescence labelling

Mesothelial EVs were incubated in the presence of 10 μM highly lipophilic nature of dyes DiO (Invitrogen, V‐22886) or PKH26 (Sigma‐Aldrich, MINI26) for 30 min at 37°C. Then the EVs were washed with PBS and ultracentrifuged again at 100,000 × *g* for 90 min to remove remaining dyes. Labelled EVs were resuspended in 100 μL of PBS and then transferred into recipient mice (100 μg per mice) or fibroblasts (30 μg/mL).

### 2.14 Immunofluorescence staining

For cellular immunofluorescence. Firstly, media was removed and coverslips were washed with PBS. Next, cells were fixed in 4% formaldehyde for 15 min. After rinsed with PBS and permeabilized with 0.3% TritonX‐100 for 10 min, cells were blocked for 1 h. Subsequently, blocking buffer were removed and coverslips were incubated with primary antibody at 4°C overnight. The next day, coverslips were washed with PBS and incubated with secondary antibody for 1 h. After washing with PBS, Coverslips were mounted in glass slides with antifade mounting medium with DAPI (Beyotime, P0131).

For immunofluorescence of frozen sections. Before staining, the frozen sections were rewarmed at room temperature for 20 min and permeabilized in 0.3% TritonX‐100 for 10 min. After delineating the tissue area with a hydrophobic pen, sections were blocked and stained as abovementioned.

The primary antibodies were used in immunofluorescence assay: anti‐CD81 (CST, 10037S, 1:100), anti‐Cytokeratin 7 (Santa Cruz, sc‐23876, 1:50), anti‐Fibronectin (Abcam, ab2413, 1:200), anti‐COL1A1 (CST, 72026S, 1:100), anti‐α‐SMA (Sigma‐Aldrich, A5228, 1:200), anti‐Vimentin (CST, 5741S, 1:200), anti‐ILK (Abcam, ab76468, 1:100). The secondary antibodies: anti‐rabbit Alexa Fluor 488 (CST, 4412S, 1:1000), anti‐mouse Alexa Fluor 488 (4408S, 1:1000), anti‐rabbit Alexa Fluor 555 (4413S, 1:1000), anti‐rabbit Alexa Fluor 594 (8889S, 1:1000), anti‐mouse Alexa Fluor 594 (8890S, 1:1000).

### 2.15 Mouse model and sample collection

All animals were fed in the Laboratory Animal Centre of South China Agricultural University. All animal procedures were approved by the Ethics Committee of South China Agricultural University (2022D079) and followed the animal research reporting of in vivo experiments (ARRIVE) guideline. Eight‐week‐old male C57BL/6 mice were purchased to make the PD‐related peritoneal fibrosis model. To induce the peritoneal fibrosis, intraperitoneal injection of 4.25% PD fluid (0.1 mL/g, daily) was given for six weeks. To determine the role of mesothelial EVs in peritoneal fibrosis, mice were randomly divided into three groups: Saline, PD fluid + Control‐EVs, PD fluid + TGF‐β‐EVs. 100 μg of Control‐EVs or TGF‐β‐EVs derived from mesothelial cells were provided three time a week by intraperitoneal injection at second week. After six weeks, two mice in each group were injected 100 μg of PKH26‐labelled EVs to detect EVs transfection efficiency. Plasma and entire anterior abdominal wall of other five mice in each group were collected (n = 5 for each group). For AAV1‐mediated ILK knockdown, eight‐week‐old male C57BL/6 mice were randomly divided into three groups: Saline, PD fluid + AAV1‐Control, PD fluid + AAV1‐shILK. AAV1 viruses (WZ biosciences) were pretreated before injection of PD fluid (1 × 10^14^ gv/Kg body weight). The mice injected AAV1‐Control viruses were detected transfection efficiency of viruses on peritoneum. After six weeks, mice were euthanized and samples were collected (n = 6 for each group). For AAV1‐mediated Rab27a knockdown, similar approach was performed (n = 6 for each group), but one mouse in AAV1‐shRab27a group was died during experiment.

### 2.16 Histology and immunohistochemical staining

Masson trichrome staining and immunohistochemical staining were performed as described previously (Si et al., 2019). Peritoneal tissues were fixed in 4% paraformaldehyde for at least 24 h at room temperature. Fixed tissues were embedded in paraffin and sectioned at 4 μm for staining. The primary antibodies used for immunohistochemical staining were anti‐Fibronectin (Abcam, ab2413, 1:200), anti‐COL1A1 (CST, 72026S, 1:100), anti‐α‐SMA (Servicebio, GB111364, 1:50), anti‐PDGFRB (CST, 3169S, 1:100), anti‐ILK (Abcam, ab76468, 1:100).

### 2.17 Peritoneal permeability test

Modified peritoneal equilibration tests were performed to detect peritoneal permeability. About 3 mL of 4.25% PD fluid was injected intraperitoneally. The dialysate and blood samples were collected 2 h later. Then, the ratio of glucose concentration of 2 h peritoneal dialysate and 0 h peritoneal dialysate (D/D0 glucose) and ratio of creatinine concentration in dialysate and in plasma (D/P Cre) at 2 h were calculated.

### 2.18 Flow cytometry of bead‐bound EVs

To evaluate the proportion of ILK^+^ EVs by flow cytometry, 1 × 10^10^ EVs resuspended in 200 μL of PBS were incubated with 20 μL of sulphate beads (Invitrogen, A37304) for 15 min in rotation. Subsequently, 400 μL of PBS was added to each sample, mixed evenly, and rotated at 4°C for the night. The next day, 300 μL of 1 M glycine was added and incubated at room temperature rotating for 1 h. After centrifugation at 14,000 × *g* for 2 min, the supernatant was discarded, and the precipitate was resuspended in 100 μL of 10% BSA and blocked for 1 h. Then the samples were centrifuged at 14,000 × *g* for 2 min, and the precipitate was resuspended in 100 μL of fixation/permeabilization reagent (eBioscience, 88‐8824‐00) for 30 min. Subsequently, the bead‐bound EVs were washed using 200 μL of permeabilization buffer and incubated with anti‐ILK antibody (Abcam, ab76468) for 1 h on ice. Then the samples were washed two times in 200 μL of permeabilization buffer and incubated with secondary antibodies (CST, 4412S). After staining, the bead‐bound EVs were resuspended in 300 μL of permeabilization buffer for flow cytometry. Data were analysed in FlowJo software.

### 2.19 ELISA for levels of cytokine

Levels of TGF‐β1 in PLF and PD effluent were determined by ELISA Kits (MEIMIAN, MM‐0090H1). All procedures were performed according to the manufacturer's instruction.

### 2.20 Statistical analysis

Data are presented as means ± SD and analysed using Prism 8 (GraphPad, Inc.) and RStudio software (version 3.6.3, RStudio Inc.). Statistical analysis was performed using the unpaired Student's *t* tests for comparing two selected groups. ANOVA methods, followed by Bonferroni's multiple comparison tests were used to compare more than two groups. Spearman order correlation analysis was used to evaluate the relationship between different factors. A value of *P* < 0.05 was considered statistically significant.

## 3 RESULTS

### 3.1 Characterization of the EVs isolated from PD effluent

**FIGURE 1:**
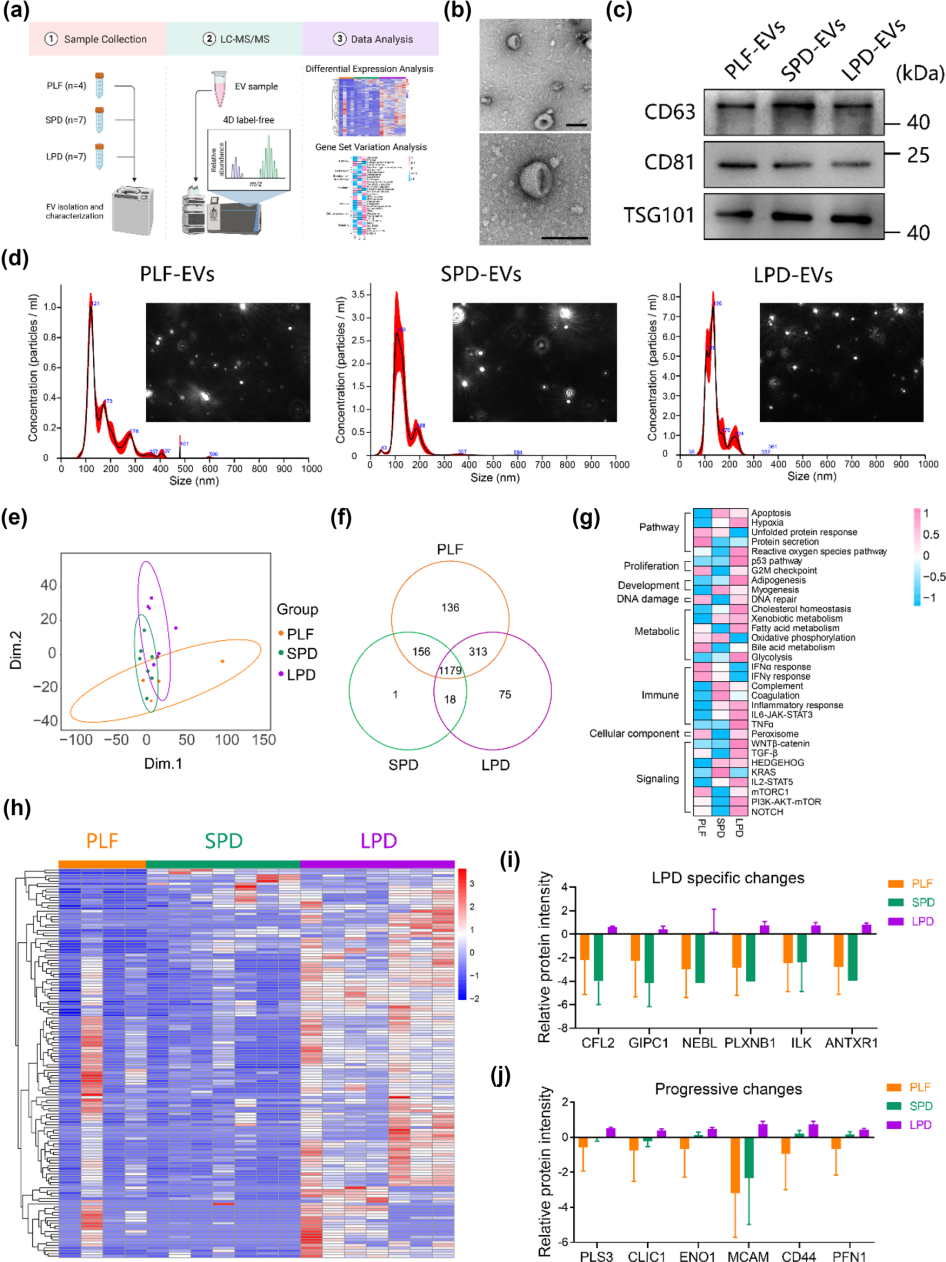
Characterization of the EVs isolated from PLF and PD effluent. (a) Graphic illustration of the workflow for isolation and proteomic analyses of effluent‐derived EVs. PLF was collected from inguinal hernia patients with normal renal function (*n* = 4). SPD was obtained from patients undergoing short‐term PD (*n* = 7). LPD was obtained from patients undergoing long‐term PD (*n* = 7). After EV isolation by ultracentrifugation, protein levels were measured and quantified using 4D label‐free mass spectrometry and analysed using GSVA and differential expression analysis. (b) Representative electron micrograph of the EVs. Scale bars, 200 nm. (c) Western blot analysis of EV classical markers CD63, CD81, TSG101 in the isolated EVs. (d) NTA showing the particle concentration and size distribution of the three groups of EVs. (e) PCA of the total EV samples. PLF, orange symbols; SPD, green symbols; LPD, purple symbols. (f) Venn diagram showing a total of 1784 proteins identified in PLF group, 1354 proteins identified in SPD group and 1585 proteins identified in LPD group. (g) GSVA of total proteins based on Hallmark gene set. (h) Heatmap showing a cluster of 134 up‐regulated DEPs in LPD‐EVs. (i) List of several DEPs that have LPD specific changes compared with PLF or SPD group. (j) List of several DEPs that have progressive changes across the comparisons of PLF, SPD and LPD groups.

The EVs were isolated from 400 mL of PLF (*n* = 4), SPD (*n* = 7) and LPD (*n* = 7) by differential ultracentrifugation (Figure 1a). The clinical information for all cases was provided in Supplementary Table S2. We validated that isolated EVs exhibited the cup‐shaped morphology using transmission electron microscopy (TEM) (Figure 1b). Western blot analysis showed that the EVs were positive for conventional EV‐specific markers including CD63, CD81, and TSG101 (Figure 1c). In addition, the three groups of EVs had a diameter range of 30–200 nm as determined by NTA (Figure 1d). In comparison to SPD, more EV particle number and protein amount were obtained from the same volume of effluent in LPD (Figure S1A–C).

Proteins packaged in EVs are involved in regulating specific cellular function under numerous physiologic and pathologic conditions (Shuen et al., 2022). To investigate the distinctive proteomic profile of effluent‐EVs from patients undergoing PD and their potential clinical applications, we performed 4D label‐free quantitative liquid chromatography‐tandem mass spectrometry (LC–MS/MS) proteomics analyses on these EVs. Principal Component Analysis (PCA) indicated a marginal separation of the three groups based on protein abundance (Figure 1e). A total of 1878 proteins were identified and analysed by Gene Set Variation Analysis (GSVA) based on Hallmark gene set (Figure 1f and g). Notably, several signalling pathways relevant to fibrosis, such as Wnt‐β‐catenin, TGF‐β and hedgehog signalling, were mostly enriched in LPD group. Moreover, the metabolism pathways involving in glycolysis and fatty acid metabolism were also enriched in LPD group, whereas the oxidative phosphorylation and bile acid metabolism pathways were enriched in both PLF and SPD group. Volcano plots showed the significantly differentially expressed proteins (DEPs) among the three groups (Figure S2A–C). There were 740 significantly altered proteins between PLF and SPD, 146 significantly altered proteins between PLF and LPD, 413 significantly altered proteins between SPD and LPD. Gene Ontology (GO) analysis revealed that these downregulated DEPs in LPD group were related to the antigen processing and presentation, and amino acid metabolic process, while the upregulated DEPs were associated with the actin polymerization or depolymerization, cytoskeletal regulation, and cell substrate adhesion (Figure S2D and E). To identify long‐term PD signature proteins, unsupervised hierarchical clustering of these DEPs was performed and a cluster of 143 DEPs was upregulated in LPD‐EVs compared to PLF or SPD‐EVs (Figure 1h). Several LPD‐specific DEPs were showed in Figure 1i, including cofilin 2 (CFL2), nebulette (NEBL), GIPC PDZ domain containing family member 1 (GIPC1), plexin B1 (PLXNB1), integrin linked kinase (ILK), ANTXR cell adhesion molecule 1 (ANTXR1), most of which have been reported to associated with cytoskeleton remodelling, fibrosis, and angiogenesis. Interestingly, several DEPs including plastin 3 (PLS3), chloride intracellular channel 1 (CLIC1), enolase 1 (ENO1), melanoma cell adhesion molecule (MCAM), CD44, profilin 1 (PFN1) showed a progressive increase across PLF, SPD, and LPD (Figure 1j). In summary, effluent‐derived EVs from patients undergoing PD with different durations presented distinct proteomic profiling, and the signature proteins in LPD‐EVs suggested their involvement in peritoneal fibrogenesis.

### 3.2 Long‐term PD stimulates the secretion of EVs in mesothelial cells

**FIGURE 2:**
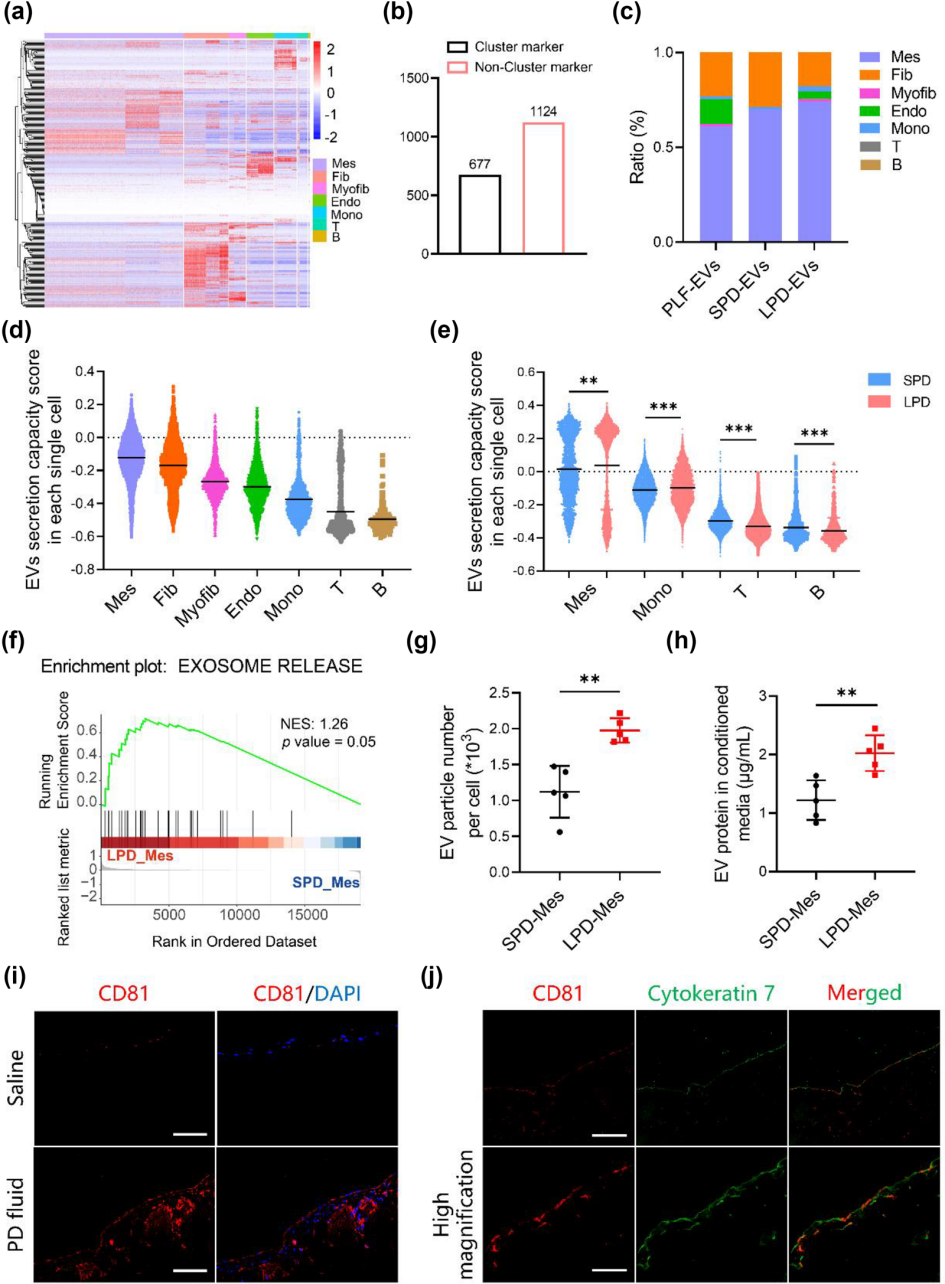
Long‐term PD stimulates the secretion of EVs in mesothelial cells. (a) Heatmap showing the expression levels of genes encoding EV proteins in each type of peritoneal cells. (b) Number of the genes encoding EV proteins identified to be “Cluster marker” or “Non‐Cluster marker”. (c) Cell origin proportion of the EVs derived from PLF, SPD or LPD. (d and e) ssGSEA scores showing EVs release capacity of each single cell in scRNA‐seq datasets of normal peritoneum (d) or PD effluent‐derived peritoneal cells (e). (f) GSEA was performed focusing on the gene set of EVs release in SPD‐Mes and LPD‐Mes group. (g) EV particle number per mesothelial cell was determined by NTA. (h) EV protein in conditioned media of mesothelial cells was measured by BCA. (i) Immunofluorescence staining showing CD81 expression in mouse peritoneum treated with PD fluid or saline. Scale bars, 100 μm. (j) Representative images of co‐staining of CD81 (red) and Cytokeratin 7 (green) at low and high magnification. Scale bars, 100 and 20 μm. **P < 0.01, and ***P < 0.001.

EVs separated from body fluids are released by various human cells and the molecular composition of EVs can reflect their origins (Shao et al., 2018). Combining proteomic data of PD effluent‐derived EVs and scRNA‐seq data of peritoneal tissues, we attempted to identify the cell origin of PD effluent‐derived EVs. Peritoneal cells in normal peritoneum were assigned to seven distinct cell types according to our previous classification criteria (Figure S3A and B). We found that the expression levels of genes encoding EV proteins in scRNA‐seq data have considerable cell‐type specificity (Figure 2a). From the total 1801 identified genes, 677 were overlapped with “Cluster markers” of the scRNA‐seq data, which differentially expressed in either cell type (log (fold change) > 0.25, min.pt = 0.2, *P*‐adj < 0.05), while others were overlapped with genes broadly expressed among all cell types, called “Non‐Cluster markers” (Figure 2b). We then evaluated the proportion of different cell type original EVs by deconvolution analysis of EV proteomics. MUlti‐Subject SIngle Cell deconvolution (MuSiC) algorithm estimated the cell type abundance using the scRNA‐seq data above as reference. The results indicated that mesothelial EVs accounted for the majority of PD effluent‐derived EVs and its proportion increased in LPD group compared to PLF or SPD group (Figure 2c).

To evaluate the ability of each single cell to release EVs, we further used all the genes encoding EV proteins as a reference gene set, representing EVs release ability, to perform a single sample Gene Set Enrichment Analysis (ssGSEA) in scRNA‐seq data of normal peritoneum. We found that parenchymal cells, especially mesothelial cells and fibroblasts, released more EVs compared to other peritoneal cells according to the ssGSEA score (Figure 2d). Similarly, in the scRNA‐seq data of PD effluent‐derived peritoneal cells (Figure S3C and D), mesothelial cells were determined to have the strongest capacity to release EVs than other cells. More importantly, mesothelial cells in LPD group held an increased EVs secretion capacity score compared to SPD group (Figure 2e). Using another enrichment analysis, in which genes related to EVs secretion were used as the reference gene set (Yang et al., 2019), we found that mesothelial cells from LPD had a significant enrichment score compared to SPD (Figure 2f). We then obtained and cultured mesothelial cells from PD effluent. EVs derived from these mesothelial cells were purified from conditioned media through ultracentrifugation and analysed by NTA (Figure S4A) and TEM (Figure S4B). More EVs, as determined by both EV particle number (Figure 2g) and the EV protein level (Figure 2h), were released from the same numbers of mesothelial cells from LPD compared with them from SPD. We also verified that the mRNA expression levels of EV biogenesis or release‐associated genes upregulated in mesothelial cells from LPD compared with SPD (Figure S5A).

To test whether PD fluid can stimulate the production of peritoneal EVs in mice, we made a mouse model of peritoneal fibrosis by daily injection of PD fluid containing 4.25% glucose for six weeks. The peritoneal tissues from mice treated with PD fluid showed increased submesothelial collagen deposition and protein expression level of α‐SMA compared to saline group (Figure S6A). We evaluated the expression levels of proteins related to EV biogenesis, including CD81 and TSG101, which were upregulated in peritoneal tissue of PD fluid group (Figure 2i and Figure S7A–C). We then investigated the source of EVs in mouse peritoneum by co‐staining of CD81 (EVs marker) and Cytokeratin 7 (mesothelial marker). The result confirmed their co‐localization, suggesting mesothelial cell was one of the sources of peritoneal EVs in mice (Figure 2j).

### 3.3 Mesothelial EVs mediate fibroblast activation in vitro

**FIGURE 3:**
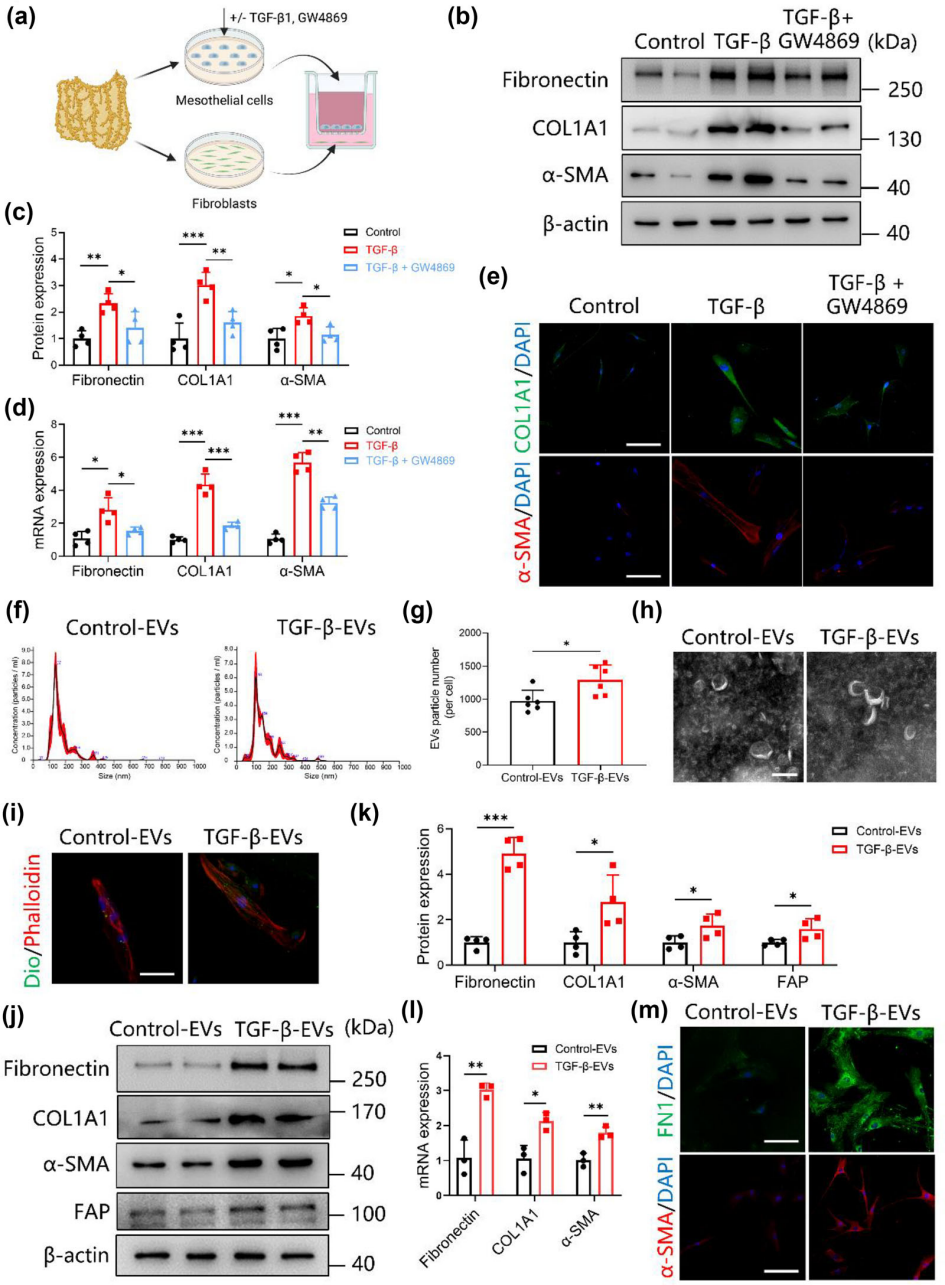
Mesothelial EVs promote fibroblast activation in vitro. (a) Schematic diagram of co‐culture of mesothelial cells and fibroblasts using a transwell system. (b‐d) Western blot (b and c) and real‐time PCR (d) analysis of fibronectin, COL1A1 and α‐SMA in fibroblasts co‐cultured with mesothelial cells. (e) Representative immunofluorescence images of COL1A1 and α‐SMA in fibroblasts. Scale bars, 100 μm. (f and g) NTA showing the size distribution and particle number of mesothelial EVs. (h) Transmission electron micrograph of mesothelial EVs. Scale bars, 100 nm. (i) Fibroblasts are incubated with DiO‐labelled mesothelial EVs for 12 h, and representative immunofluorescence images show the successful delivery of DiO‐labelled EVs (green) into Phalloidin‐labelled fibroblasts (red). Scale bars, 50 μm. (j and k) Representative western blot (j) and quantification (k) of protein expression of fibronectin, COL1A1, α‐SMA and FAP in fibroblasts treated with mesothelial EVs. (l) Real‐time PCR analysis of fibronectin, COL1A1 and α‐SMA mRNAs in fibroblasts after incubation with mesothelial EVs. (m) Representative micrographs showing immunofluorescence staining of fibronectin and α‐SMA in fibroblasts incubated with mesothelial EVs. Scale bars, 100 μm. *P < 0.05, **P < 0.01, and ***P < 0.001.

Previous studies have reported that fibroblast activation plays a central role in the progression of peritoneal fibrosis. We inquired whether mesothelial EVs contribute to the activation of fibroblasts in vitro. To address this question, human primary peritoneal mesothelial cells and fibroblasts were separated from normal peritoneum. The primary mesothelial cells were treated with transforming growth factor‐β1 (TGF‐β1) for 24 h to simulate the condition of mesothelial cells in injured peritoneum. After thoroughly washing, these cells were then co‐cultured with primary peritoneal fibroblasts in a transwell system, whereby mesothelial cells did not physically interact with fibroblasts (Figure 3a). After 3 days of co‐culture, these fibroblasts exhibited higher expression of mRNAs and proteins related to fibroblast activation compared with fibroblasts co‐culture with non‐treated mesothelial cells. More importantly, blocking mesothelial EVs synthesis/secretion by GW4869, a noncompetitive phospholipase inhibitor, markedly inhibited fibroblast activation and extracellular matrix (ECM) production (Figure 3b–e). Similarly, we also observed increased expression of fibroblast activation related genes in fibroblast cell line HFF‐1 after they co‐cultured with TGF‐β1‐pretreated mesothelial cell line MeT5A. Blockade of mesothelial EVs synthesis/secretion significantly suppressed fibroblast activation induced by co‐culture with pretreated MeT5A cells, suggesting that mesothelial EVs participated in fibroblast activation (Figure S8A–H).

To confirm that mesothelial EVs directly activate fibroblasts, we separated EVs (Control‐EVs or TGF‐β‐EVs) from the supernatant of mesothelial cells stimulated with or without TGF‐β1 and determined the size distribution and concentration of EVs using NTA (Figure 3f). Mesothelial EVs were mostly distributed in the range of 30–200 nm and their amount were significantly enhanced by TGF‐β1 treatment. Subsequently, the cup‐shaped morphology was confirmed by TEM and positive EVs biomarkers were also evaluated by western blot (Figures 3h and S9A). To examine whether fibroblasts can uptake mesothelial EVs, we labelled the EVs with DiO dyes and incubated them with fibroblasts. After 12 h, mostly of the EVs were visualized in fibroblasts (Figure 3i). As expected, these TGF‐β1‐stimulated EVs markedly increased the expression of mRNAs and proteins related to activation and ECM production in fibroblasts (Figure 3j–m). Collectively, these data demonstrated that mesothelial cells promote fibroblast activation via EVs transportation.

### 3.4 Mesothelial EVs promote peritoneal fibrosis in vivo

**FIGURE 4:**
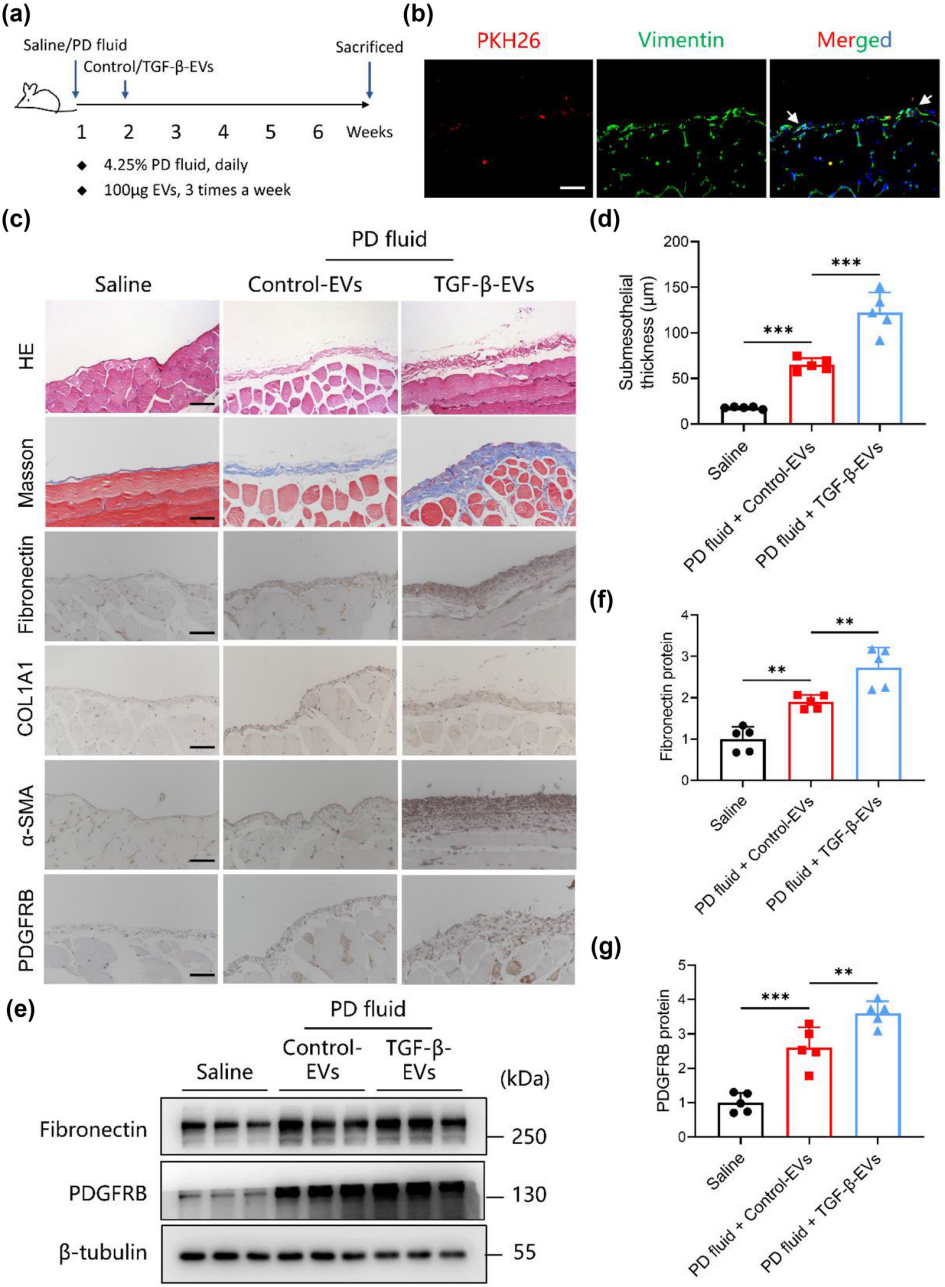
Mesothelial EVs aggravate peritoneal fibrosis in vivo. (a) Schematic diagram of experimental design. Arrows denote the time point of 4.25% PD fluid injection (0.1 mL/g, daily), mesothelial EVs treatment (100 μg, 3 times/week), and peritoneum samples collection. (b) Representative images showing the uptake of PKH26‐labelled EVs (red) in mouse peritoneal fibroblasts (vimentin, green). Arrows indicate co‐localization of mesothelial EVs with fibroblasts. Scale bars, 50 μm. (c) Representative HE staining, Masson staining and fibronectin, COL1A1, α‐SMA and PDGFRB staining views of parietal peritoneal tissues in saline (*n* = 5), PD fluid + Control‐EVs (*n* = 5) and PD fluid + TGF‐β‐EVs groups (*n* = 5). Scale bars, 50 μm. (d) Quantitation of peritoneal thickness. (e‐g) Representative western blot (e) and quantification (f–g) of protein expression of fibronectin and PDGFRB in peritoneal tissues. **P < 0.01 and ***P < 0.001.

We next aimed to determine the role of mesothelial EVs in regulating peritoneal fibrogenesis in mice. To this end, we isolated Control‐EVs or TGF‐β‐EVs from cultured mesothelial cells treated with or without TGF‐β1 and examined how these EVs influence the fibrogenesis induced by PD fluid. The experimental design and treatment duration were shown in Figure 4a. At the second week of modelling, PD fluid‐treated mice were given same amounts of Control‐EVs or TGF‐β‐EVs (100 μg, 3 times/week) by intraperitoneal injection for 5 weeks. To test efficient uptake of mesothelial EVs in mouse peritoneal fibroblasts, we performed co‐staining of PKH‐26 labelled mesothelial EVs and fibroblast marker vimentin. As shown in Figure 4b, PKH‐26 fluorescence (red) and vimentin (green) were co‐localized in peritoneum, confirming the uptake of EVs.

We then evaluate the effects of EV treatments on the development of peritoneal fibrosis. Histology examination revealed that PD fluid caused significant thickening of parietal peritoneum in both Control‐EVs and TGF‐β‐EVs treated mice compared with saline‐treated mice, as evaluated by 6 independent measurements at standardized interspaced locations on the peritoneum. Immunohistochemical staining showed an increase in α‐SMA expression that was associated with fibronectin and collagen deposition in fibrotic peritoneum of mice treated with Control‐EVs, however, these responses were aggravated by injection of TGF‐β‐EVs (Figure 4c and d). Consistent with these observations, western blotting demonstrated that the protein expression of fibronectin and PDGFRB were significantly increased in the fibrotic peritoneum of mice treated with TGF‐β‐EVs compared with the results of Control‐EVs‐treated mice (Figure 4e–g). These results indicated that mesothelial EVs promote the progression of peritoneal fibrosis by directly activating peritoneal fibroblasts.

### 3.5 Blocking EVs secretion attenuates PD fluid‐induced fibrogenesis

**FIGURE 5:**
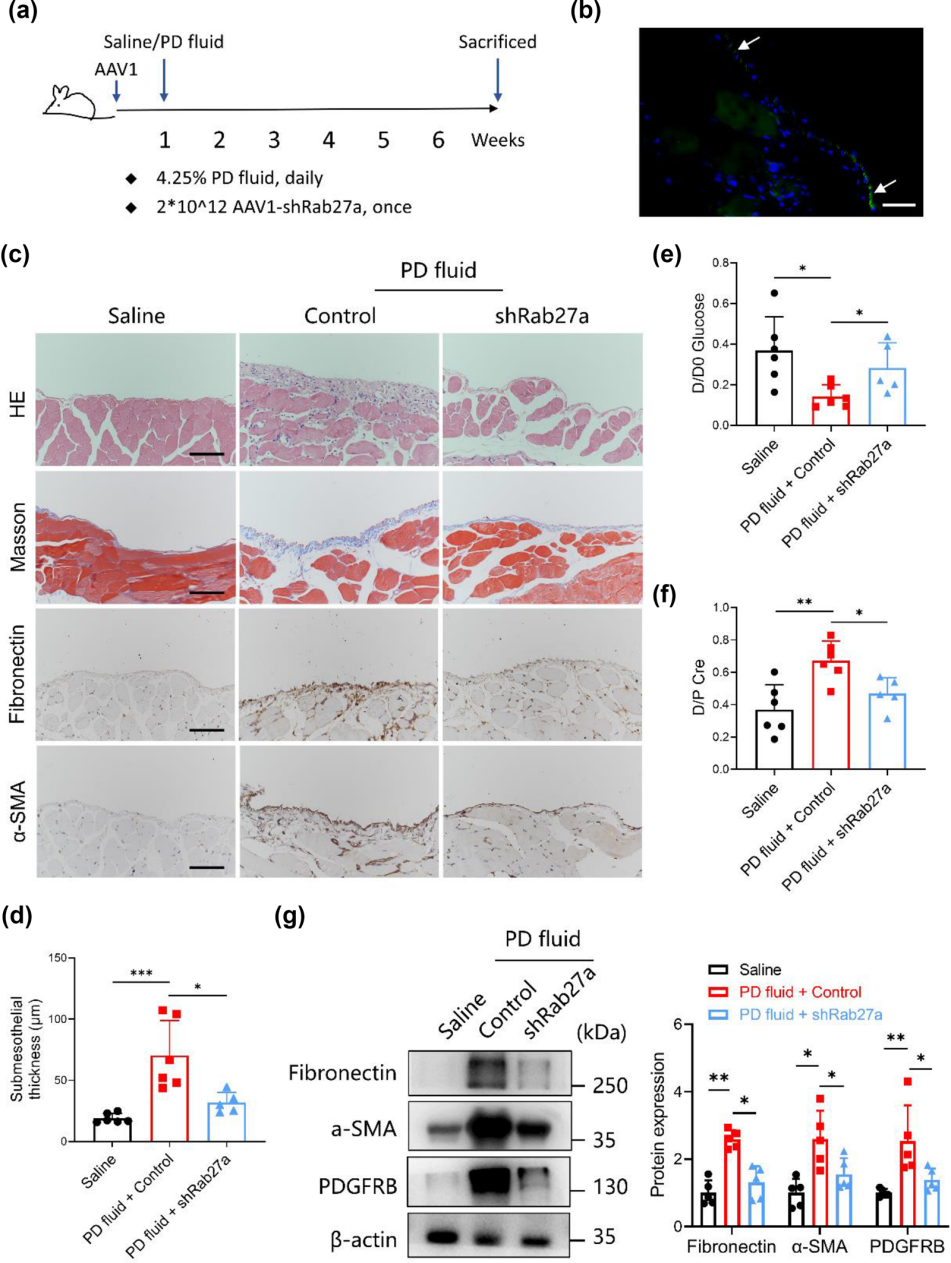
Blocking EVs secretion attenuates PD fluid‐induced peritoneal fibrogenesis. (a) Schematic for the AAV‐shRab27a and AAV‐Control administration protocol. (b) Representative image of mouse peritoneum transfected with AAV‐GFP. Arrows indicate the delivery of AAV (green) to peritoneal cells. Scale bars, 50 μm. (c) Representative HE staining, Masson staining and fibronectin and α‐SMA staining images of peritoneum in saline (*n* = 6), PD fluid + AAV1‐Control (*n* = 6) and PD fluid + AAV1‐shRab27a (*n* = 5) groups. Scale bars, 50 μm. (d) Quantitation of peritoneal thickness of mice. (e and f) Peritoneal permeability of mice examined by modified peritoneal equilibration test. (g) Representative western blot and quantification analysis of protein expression of fibronectin, α‐SMA and PDGFRB in peritoneal tissues. *P < 0.05, **P < 0.01, and ***P < 0.001.

Given that blocking EVs secretion in cultured mesothelial cells suppress fibroblast activation in vitro, we subsequently tested whether impairment of EVs secretion could inhibit fibroblast activation and the development of fibrosis in a mouse model of PD fluid‐induced peritoneal fibrosis. Since small GTPase Rab27a reportedly regulates the exocytosis process of vesicles of endosomal origin (Ostrowski et al., 2010), we generated Adeno‐associated viruses 1 (AAV1s) encoding short hairpin RNA (shRNA) that targets Rab27a to block EVs secretion. As shown in Figure 5a, we injected AAV1‐shRab27a or AAV1‐carrying scramble shRNA‐GFP (interperitoneally, 1 × 10^14^ gv/Kg) into mice one week before PD fluid treatment. Using AAV1‐scramble shRNA‐GFP, we evaluated the transfection efficiency of AAV1 in peritoneal tissue. 72 h after injection of the virus, GFP fluorescence was clearly observed in nearly 100% of peritoneal cells (Figure 5b). At the end of PD fluid treatment, mice injected with AAV1‐scramble shRNA developed distinct peritoneal thickness and fibrosis. In contrast, injection of AAV1‐shRab27a markedly attenuated PD fluid‐induced thickening of mouse peritoneum (Figure 5c). Immunostaining also revealed a reduction of fibronectin and α‐SMA in fibrotic peritoneum after Rab27a knockdown (Figure 5d). Peritoneal dysfunction is a typical feature of peritoneal fibrosis and can be assessed by modified peritoneal equilibration test, which concludes the measurement of the ratio of glucose concentration of 2 h peritoneal dialysate and 0 h peritoneal dialysate (D/D0 glucose) and the ratio of creatinine concentration in dialysate and plasma (D/P Cre). We recorded a high peritoneal permeability in mice treated with PD fluid + AAV1‐Control, while the peritoneal dysfunction was significantly improved by Rab27a knockdown (Figure 5e and f). Western blot analysis also showed that the induction of fibronectin, α‐SMA and PDGFRB in fibrotic peritoneum was significantly reduced by knockdown of Rab27a (Figure 5g). Collectively, these findings indicated that blockade of EVs secretion significantly alleviates PD fluid‐induced peritoneal fibrosis in mice.

### 3.6 ILK is enriched in mesothelial EVs and promotes fibroblast activation via p38 MAPK signalling pathway

**FIGURE 6:**
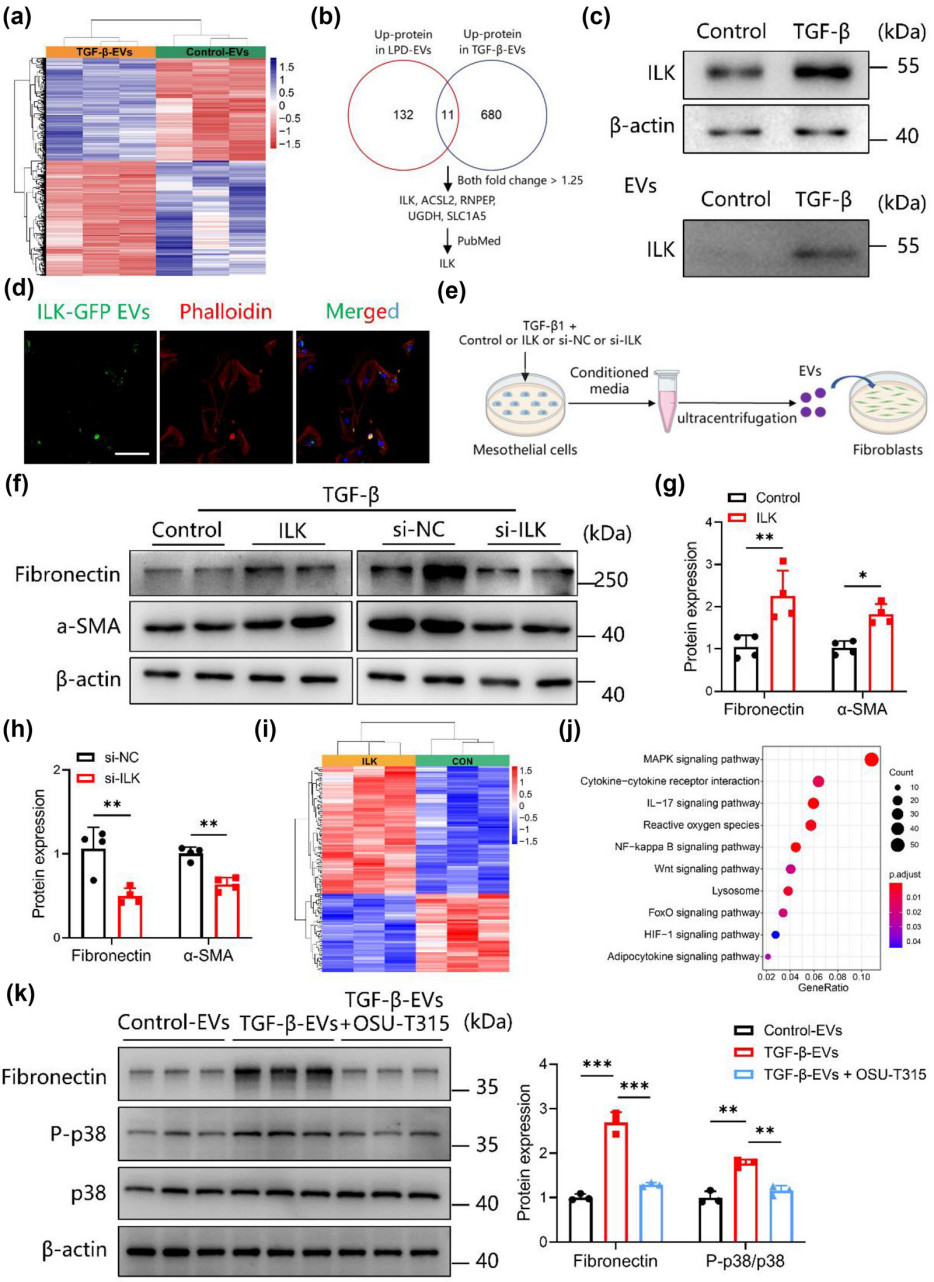
ILK is enriched in mesothelial EVs and promotes fibroblast activation via p38 MAPK signalling pathway. (a) Heatmap showing the differential expression of proteins between Control‐EVs and TGF‐β‐EVs (*n* = 3). (b) The schematic of screening target proteins. (c) Western blot analysis of ILK protein expression in mesothelial cells and mesothelial EVs. (d) Imaging of cytoskeleton of fibroblasts (red) after incubation with the EVs derived from mesothelial cells pre‐transfected with ILK‐GFP plasmid (green). Scale bars, 100 μm. (e) Experimental design showing overexpression or knockdown of ILK in mesothelial cells prior to EV isolation from conditioned media. (f‐h) EVs were isolated from mesothelial cells transfected with ILK overexpression plasmid or ILK‐siRNA, and then incubated with fibroblasts. Representative western blot (f) and quantification (g and h) analysis of protein expression of fibronectin and α‐SMA in fibroblasts were showed. (i) Gene expression profiling by RNA‐seq shows differential gene clustering of fibroblasts treated with or without ILK plasmid (*n* = 3). (j) Bubble diagram showing the enrichment of pathways. (k) Representative western blot and quantification analysis of protein expression of P‐p38, p38 and fibronectin in fibroblasts treated with Control‐EVs, TGF‐β‐EVs, or TGF‐β‐EVs + OSU‐T315 (a small molecule inhibitor of ILK). *P < 0.05, **P < 0.01, and ***P < 0.001.

To explore mechanisms by which mesothelial EVs promote fibroblast activation and peritoneal fibrogenesis, we performed 4D label‐free proteomic analyses of Control‐EVs and TGF‐β‐EVs derived from mesothelial cells. A total of 3821 proteins were captured and the differential proteins between two groups were showed in Figure 6a. To identify the effective component regulating fibroblast activation, we overlapped this result with the proteomics dataset obtained in PD effluent‐derived EVs. We found that five proteins, including ILK, acyl‐CoA synthetase long chain family member 2 (ACSL2), arginyl aminopeptidase (RNPEP), UDP‐glucose 6‐dehydrogenase (UGDH), solute carrier family 1 member 5 (SLC1A5), were upregulated in both TGF‐β‐EVs and LPD‐EVs (Figure 6b). Since ILK has been reportedly linked to organ fibrosis, such as renal and cardiac fibrosis (Li et al., 2009; Thakur et al., 2014), we hypothesized that this protein could be transmitted to fibroblasts, incurring fibroblast activation. To test this possibility, we first confirmed that TGF‐β1 not only stimulated the expression of ILK in primary mesothelial cells, but also increased the protein content of ILK in EVs secreted by mesothelial cells (Figure 6c). To validate whether ILK packaged in EVs can be transported to fibroblasts, we constructed a plasmid carrying ILK‐GFP fusion protein and transfected into mesothelial cells cultures. Mesothelial EVs were then isolated from the cell supernatant and added to fibroblasts. The GFP fluorescence was observed in fibroblasts 6 h after addition, indicating that ILK is efficiently transported from mesothelial cells to fibroblasts via EVs (Figure 6d). Next, we inquired whether ILK packaged in mesothelial EVs is responsible for fibroblast activation. TGF‐β1‐stimulated mesothelial cells were transfected with ILK overexpression plasmid or ILK‐siRNA. After that, EVs were isolated from these mesothelial cells and then co‐culture with fibroblasts, respectively (Figure 6e). The transfection efficiency of ILK plasmid and ILK‐siRNA were showed in Figure S10A–C. We found that EVs derived from ILK over‐expressed mesothelial cells significantly stimulated the expression of proteins related to fibroblast activation, whereas knockdown of ILK decreased the expression of these proteins in fibroblasts (Figure 6f–h).

To examine the signalling pathways which mediate fibroblast activation by ILK cargo, we performed an RNA‐seq in control fibroblasts and ILK plasmid‐infected fibroblasts (Figure 6i). Enrichment analysis of differentially expressed genes showed the significant enrichment of MAPK signalling pathway (Figure 6j), indicating that overexpression of ILK sufficiently activates MAPK signalling pathways, consistent with the observation that ILK acts as an upstream regulator of MAPK signalling (Zhang et al., 2006). Using western blot analysis, we also demonstrated that OSU‐T315, a specific small molecule inhibitor of ILK was sufficient to suppress p38 MAPK phosphorylation and the activation of fibroblasts induced by mesothelial EVs (Figure 6k), suggesting that ILK packaged in mesothelial EVs activate fibroblasts via p38 MAPK signalling pathway. Taken together, we confirmed that ILK is enriched in TGF‐β1‐stimulated mesothelial EVs and this ILK cargo promotes fibroblast activation.

### 3.7 ILK could be a promising therapeutic target for peritoneal fibrosis

**FIGURE 7:**
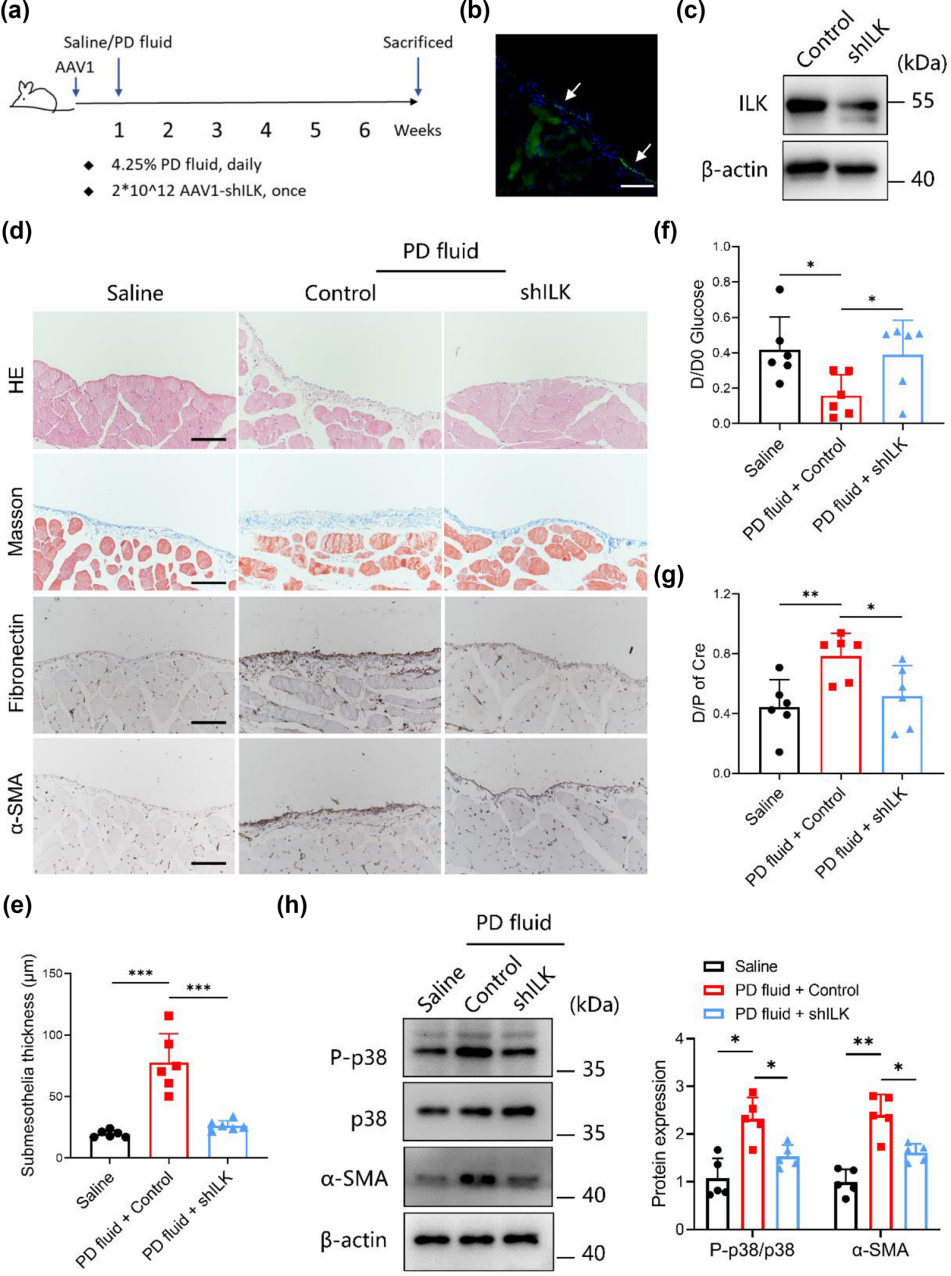
Knockdown of ILK alleviates peritoneal fibrosis in a mouse model of peritoneal dialysis. (a) Schematic diagram of experimental design. (b) Representative image of mouse peritoneum transfected with AAV‐GFP. Scale bars, 50 μm. (c) The ILK expression in peritoneal tissues detected after the mice had been treated with AAV1‐shILK. (d) Representative HE staining, Masson staining and fibronectin and α‐SMA staining images of peritoneal tissues in saline (*n* = 6), PD fluid + AAV1‐Control (*n* = 6) and PD fluid + AAV1‐shILK (*n* = 6) groups. Scale bars, 50 μm. (e) Quantitation of peritoneal thickness of the three groups. (f and g) Peritoneal permeability of mice examined by modified peritoneal equilibration test. (h) Western blot analysis showing peritoneal expression of α‐SMA, P‐p38 and p38 proteins in different groups as indicated. *P < 0.05, **P < 0.01, and ***P < 0.001.

We next sought to determine whether knockdown of ILK can alleviate mouse peritoneal fibrosis. AAV1s encoding shRNA that targets ILK were injected into abdominal cavity of mice at a week before PD fluid treatment to block the expression of ILK in mice (Figure 7a). About 72 h after injection of AAV1‐GFP, we observed that the viruses were delivered to peritoneal cells efficiently (Figure 7b). Western blot analysis also confirmed the ILK knockdown in mouse peritoneum after injection of AAV1‐shILK (Figure 7c). At the end of experiment, we examined the scar formation induced by PD fluid in peritoneum and found that the peritoneal thickening was significant decreased in AAV1‐shILK treated group compared with the result of AAV‐Control treated group. Moreover, immunostaining showed that protein expressions of fibronectin and α‐SMA were downregulated in mouse peritoneum by ILK knockdown (Figure 7d and e). As expect, PD fluid‐caused peritoneal dysfunction was also alleviated by ILK knockdown (Figure 7f and g). In addition, western blot analysis revealed that the phosphorylation level of p38 and protein expression of α‐SMA were decreased by ILK knockdown in mice given PD fluid (Figure 7h). These results support the conclusion that ILK activates p38 MAPK pathway to promote fibroblast activation. Thus, targeting ILK is a potential therapeutic strategy to prevent PD‐induced peritoneal fibrosis.

### 3.8 ILK correlates with PD‐induced fibrosis in human peritoneum

**FIGURE 8:**
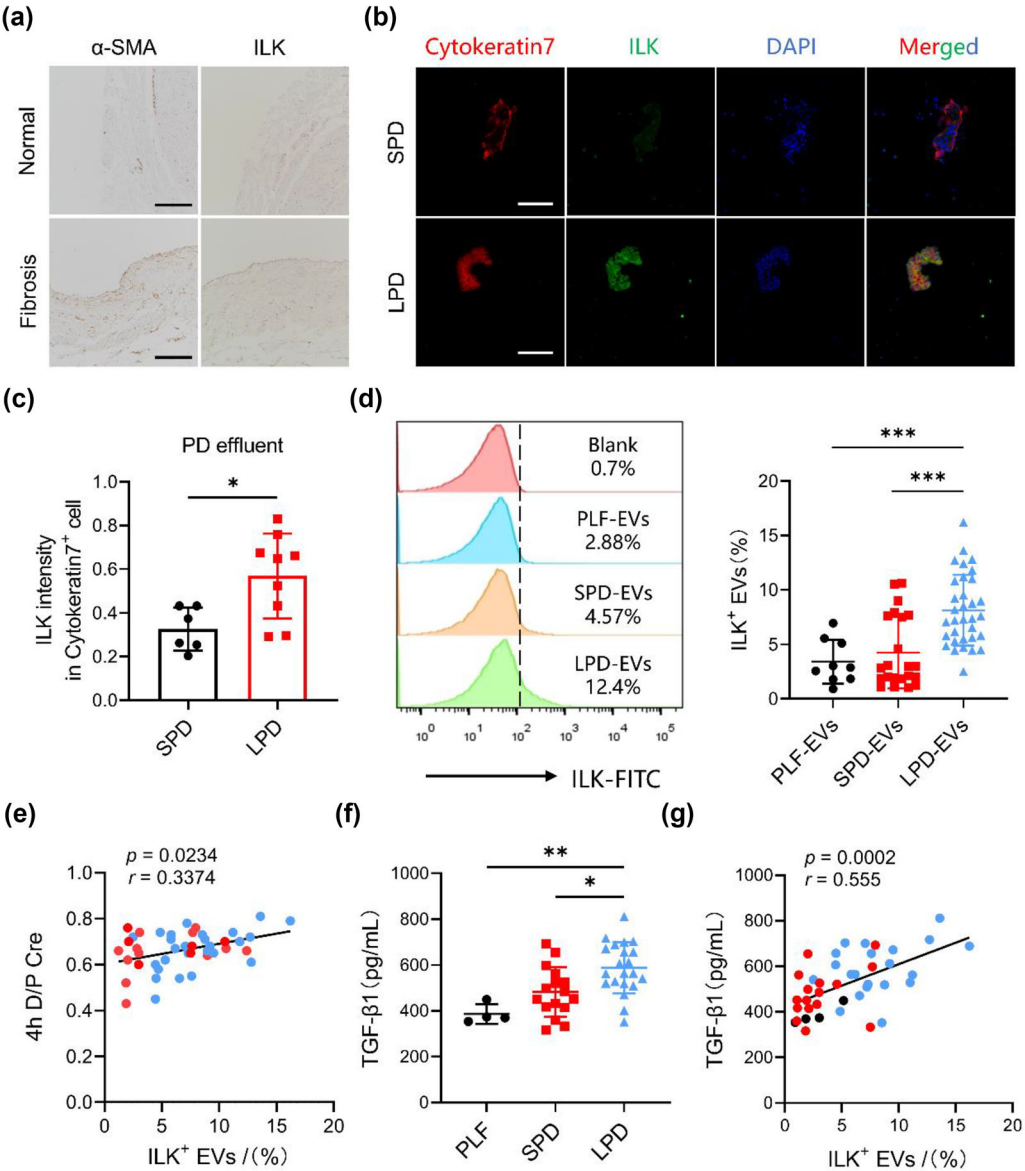
ILK correlates with PD‐induced fibrosis in human peritoneum. (a) Representative images of α‐SMA and ILK staining in human normal and fibrotic peritoneal tissues. Scale bars, 100 μm. (b and c) Representative images and quantification analysis of co‐staining of ILK and mesothelial cell marker Cytokeratin 7 in PD effluent‐derived cells. Scale bars, 100 μm. (d) FACS analysis and representative histogram plots of ILK‐positive EVs derived from PLF (*n* = 9), SPD (*n* = 22) and LPD (*n* = 33). (e) Scatter plots of the correlation between the percentage of ILK^+^ EVs and 4 h D/P Cre (*n* = 45). Red dots indicate samples in SPD group. Blue dots indicate samples in LPD group. (f) ELISA assays showing the levels of TGF‐β1 in PLF (*n* = 4), SPD (*n* = 15) and LPD (*n* = 22). (g) Scatter plots of the correlation between the percentage of ILK^+^ EVs and TGF‐β1 concentrations (*n* = 41). *P < 0.05, **P < 0.01, and ***P < 0.001.

Given that ILK in mesothelial EVs mediates the interaction between mesothelial cells and fibroblasts to promote peritoneal fibrosis in mice, we next attempted to determine whether the expression level of ILK in human peritoneum could reflect the development of peritoneal fibrosis in patients with long‐term PD. We observed that ILK was up‐regulated in fibrotic peritoneum compared with normal peritoneum (Figure 8a). In addition, we performed the co‐staining of ILK and mesothelial cell marker Cytokeratin 7 in PD effluent‐derived peritoneal cells. The percentage of ILK positive mesothelial cells increased in LPD effluent‐derived peritoneal cells compared with that in SPD (Figure 8b and c).

To validate the increased level of ILK in LPD effluent‐derived EVs, we gathered a cohort of 64 samples that were collected from nine inguinal hernia patients with normal renal function and 55 patients undergoing PD. The baseline demographics and clinical characteristics of these patients are shown in Supplementary Table S2. ILK positive EVs coupled to beads (ILK^+^ EVs) were identified in PLF and PD effluent by flow cytometry. The percentage of ILK^+^ EVs in LPD effluent was higher than these in SPD effluent or PLF (Figure 8d), suggesting that the percentage of ILK^+^ EVs was directly proportional to the duration of PD. In addition, we observed a linear correlation between the percentage of ILK^+^ EVs in PD effluent and 4 h D/P Cre (an index to evaluate the condition of peritoneal membrane in patients undergoing PD) using Spearman's correlation analysis (Figure 8e). We also evaluated the association between the percentage of ILK^+^ EVs and levels of peritoneal injury factors TGF‐β1 in PLF and PD effluent (*n* = 41). Using ELISA, we demonstrated that the level of TGF‐β1 was higher in the group that underwent long‐term PD than in the short‐term PD group (Figure 8f). Furthermore, the percentage of ILK^+^ EVs was correlated with the levels of TGF‐β1 in PLF and PD effluent (Figure 8g). These data strongly attest that an increased level of ILK is correlated with the progression of peritoneal fibrosis, which may represent a potential biomarker to predict fibrogenesis in patients undergoing PD.

## 4 DISCUSSION

The peritoneum is composed of a single layer of mesothelial cells and a sub‐mesothelium characterized by fibroblasts, adipocytes, subcollagen fibres, lymphatic vessels, and capillaries, accompanied with infiltration of scattered immune cells (Tarhan et al., 2012). During long‐term PD, the levels of various metabolic and toxic factors increase, which induce functional reprogramming in peritoneal mesothelial cells and other peritoneal cells, accompanied with ECM remodelling (Yáñez‐Mó et al., 2003; Zhang et al., 2021). In this complex microenvironment, frequent cell‐cell communications are established to adapt to the changing of environment. In addition to some soluble cytokines and chemokines, EVs are considered as part of the intercellular signalling network (Hou et al., 2020; Ying et al., 2021). The fact that EVs can be found in PD effluent offers a glimpse into the pathological state of peritoneal cells. In previous studies, effluent‐derived EVs in patients undergoing PD with high/high average transport (H/HA) or low/low average transport (L/LA) presented distinct protein expression profiles (Fang et al., 2022). Our data further demonstrated that proteins in LPD‐EVs are enriched in the pathways relevant to fibrosis and metabolism, suggesting the dynamical alteration of EVs in abdominal cavity microenvironment.

Studies have shown that PD effluent‐derived EVs are positive for the mesothelial marker mesothelin, supporting that mesothelial cells may be one of the sources of these EVs (Bruschi et al., 2021; Fang et al., 2022). However, using single cell marker to determine the source of PD effluent‐derived EVs is not sufficient, and the specific cell sources of these EVs remain largely unknown. We provided a more comprehensive assessment based on integrative analysis of PD effluent‐derived EV proteomics and scRNA‐seq data of peritoneum, and found that EVs derived from mesothelial cells account for the main part of PD effluent‐derived EVs. More interestingly, single mesothelial cell released the most EVs among fibroblasts, endothelial cells, and other immune cells. This could be due to the active metabolism, frequent endocytosis and exocytosis in mesothelial cells (Nagai et al., 2013). The production and content of EVs are determined by not only their cellular origin, but also cellular status and extracellular microenvironment. We found that long‐term PD stimulates mesothelial cells to release more EVs, which is consistent with the theory that EVs secretion could increase under the stimulation of metabolic and toxic factors and oxidative stress (Wang et al., 2021).

EVs are involved in maintaining body homeostasis and health in a physiological manner and in regulating various disease progression (Muraoka et al., 2020; Zhang et al., 2022). Increasing evidence indicated that EVs are potential active participants in tissue fibrosis via mediating intercellular communication. It has been reported that fibrotic factors in hepatocyte‐derived EVs regulate liver fibrosis by mediating hepatic stellate cells activation (Kostallari et al., 2021). Moreover, epicardial fat‐derived EVs harbour greater amounts of proinflammatory and profibrotic cytokines, and promote the development of atrial fibrillation and cardiac fibrosis (Shaihov‐Teper et al., 2021). In addition, the communication between renal tubular cells and interstitial fibroblasts via EVs transmission plays a crucial role in renal fibrosis. Reduction of EVs secretion via pharmacological inhibition or depletion of Rab27a is sufficient to attenuate renal fibrosis (Liu et al., 2020). In the present study, we demonstrated that mesothelial EVs regulate the activation of fibroblasts in PD‐induced peritoneal fibrogenesis. Specifically, blocking mesothelial EVs synthesis/secretion by GW4869 markedly attenuate the activation of fibroblasts co‐cultured with injured mesothelial cells, while TGF‐β1‐stimulated mesothelial EVs promote fibroblast activation and ECM production in fibroblasts. Using a mouse model of peritoneal fibrosis induced by PD, we confirmed that injection of TGF‐β‐EVs aggravates PD‐induced peritoneal fibrosis, while impairing EVs secretion by Rab27a knockdown leads to the alleviation of peritoneal fibrosis and improvement of peritoneal dysfunction. Considering our data, EV‐mediated intercellular communication between mesothelial cells and fibroblasts could play a critical role in the pathogenesis of peritoneal fibrosis.

Proteins can be sorted into EVs and selectively induce specific signals in recipient cells to modulate processes such as angiogenesis, apoptosis, balance of immune response, and metabolic reprogramming (Morrissey et al., 2021; Tong et al., 2021; Zhang et al., 2021). Using EV proteomics analysis, we found that ILK is enriched in both TGF‐β1‐stimulated mesothelial EVs and LPD‐EVs. Increased ILK activity links to epithelial‐mesenchymal transition production and the progression of renal fibrosis (Li et al., 2009). Small interfering RNA targeting this protein kinase is also confirmed to suppress the mesothelial‐to‐mesenchymal transition in high glucose stimulated mesothelial cells in vitro (Luo et al., 2014). Additionally, ILK is highly enriched in IL‐10KO‐endothelial progenitor cell‐derived exosomes and can activate NF‐κB pathway and downstream gene transcription in recipient endothelial cells (Yue et al., 2020). We demonstrated that ILK packaged in mesothelial EVs is effectively transferred to peritoneal fibroblasts and activates fibroblasts via p38 MAPK signalling pathway. Notably, we confirmed that ILK is up‐regulated in human fibrotic peritoneum compared to normal peritoneum, supporting the notion that ILK regulates peritoneal fibrosis, as noted in cell cultures and mice. Since peritoneal fibrosis induced by long‐term PD can lead to functional injury in the peritoneum, we then evaluated the level of ILK in PD effluent‐derived EVs and found a positive linear association between percentage of ILK^+^ EVs and 4 h D/P Cre. Moreover, the percentage of ILK^+^ EVs also correlates with the level of TGF‐β1 in PD effluent. Considering that mesothelial cells are the main source of EVs in PD effluent, we believed that these ILK^+^ EVs mainly came from mesothelial cells.

There are several limitations in our study. In addition to being released into PD effluent, EVs can also be secreted into peritoneal tissues or intercellular space. Studies focusing on EVs separated directly from human peritoneal tissues may provide more valuable information. Additionally, EVs can carry a variety of bioactive molecules containing lipids, miRNAs, mRNAs and even DNAs. We cannot rule out the potential role of other EV molecules in peritoneal fibrogenesis.

**FIGURE 9:**
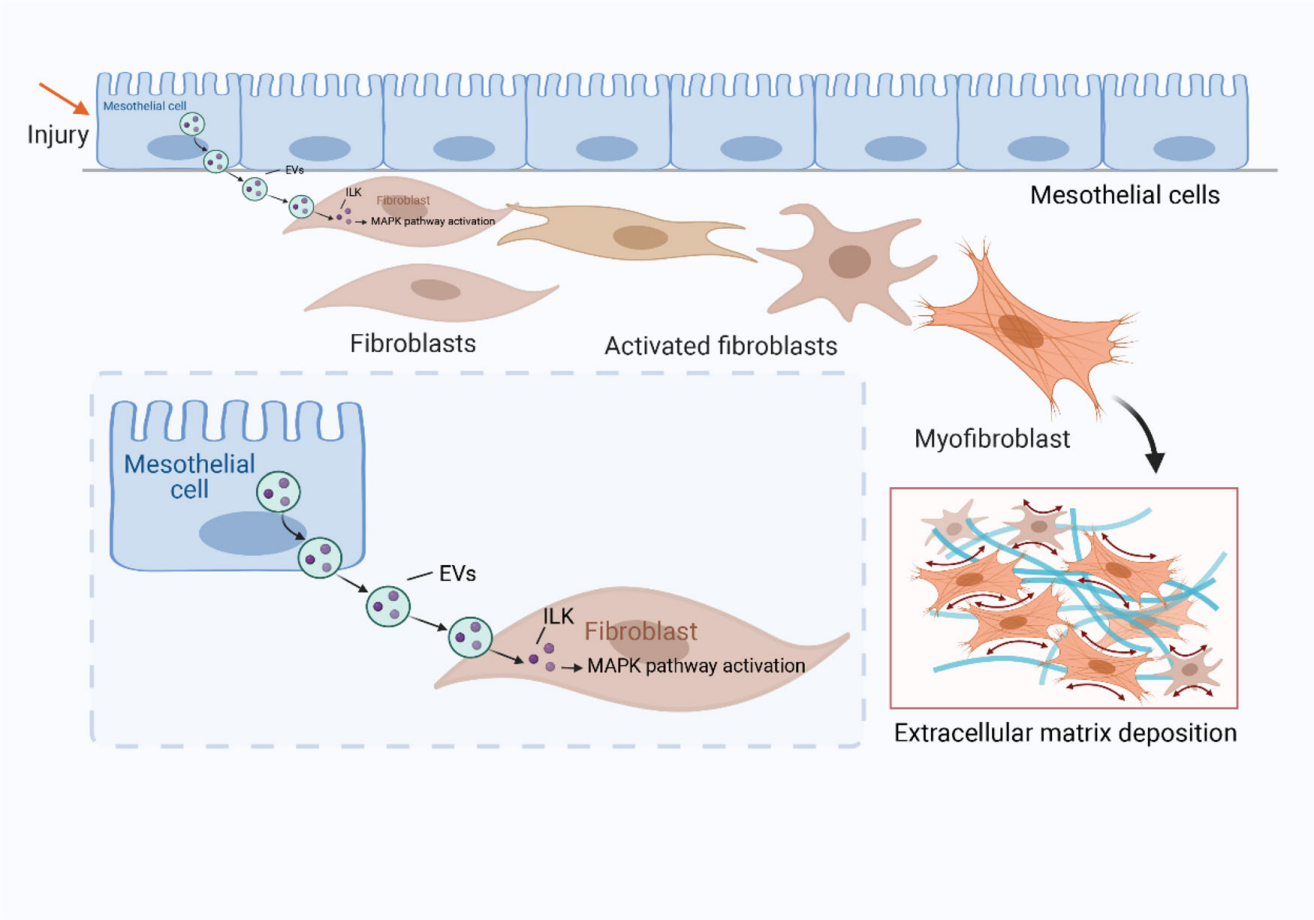
Schema depicting regulations of mesothelial EVs on fibroblast activation in peritoneum.

In summary, our data indicate that ILK‐enriched EVs derived from injured mesothelial cells activate resident fibroblasts to promote peritoneal fibrosis. ILK^+^ EVs can be used as a biomarker for peritoneal function and potential therapeutic target to treat peritoneal fibrosis (Figure 9).

## AUTHOR CONTRIBUTIONS

Qiang Huang, Hui Peng, Zengchun Ye and Zhaoyong Hu designed the study and wrote the manuscript. Qiang Huang, Yuxiang Sun, Long Peng, Juan Sun, Zixin Sha, Hongchun Lin, Yongjie Li and Canming Li performed the experiments and analyzed the data. Huiqun Li, Hongli Shang, Yanxu Chen, Xianrui Dou helped in the collection of samples from the patients.

## CONFLICT OF INTEREST STATEMENT

The authors declare that they have no competing interests.

## Supporting information

Supporting Information

Supporting Information
