# Supplementary material for: Extracellular vesicle‐packaged ILK from mesothelial cells promotes fibroblast activation in peritoneal fibrosis
Source: J Extracell Vesicles. 2023 Jun 26;12(7):12334. doi: 10.1002/jev2.12334 (PMC10291285; doi:10.1002/jev2.12334)
Supplement: Supplementary file 1 — Supporting Information [file JEV2-12-12334-s001.docx]

Supplementary Materials for

**Extracellular vesicle-packaged ILK from mesothelial cells promotes fibroblast activation in peritoneal fibrosis**

**The PDF file includes:**

Fig. S1. Yield of EVs extracted from PLF and PD effluent.

Fig. S2. Proteomic analyses of the PD effluent-derived EVs.

Fig. S3. Clustering of various cells in normal peritoneal tissues and effluent-derived peritoneal cells from patients undergoing PD.

Fig. S4. Biochemical characterization of EVs isolated from the conditioned media of mesothelial cells.

Fig. S5. The mRNA expression levels of EV biogenesis or release-associated genes upregulated in mesothelial cells from LPD.

Fig. S6. The creation of a mouse model of peritoneal fibrosis induced by PD fluid.

Fig. S7. The production of EVs increases in a mouse model of peritoneal fibrosis induced by PD fluid.

Fig. S8. Blockade of MeT-5A cell-derived EVs secretion attenuates fibroblast activation.

Fig. S9. EV markers are positive in mesothelial EVs.

Fig. S10. The overexpression and knockdown of ILK in mesothelial cells and mesothelial EVs.

**Supplementary Figures**


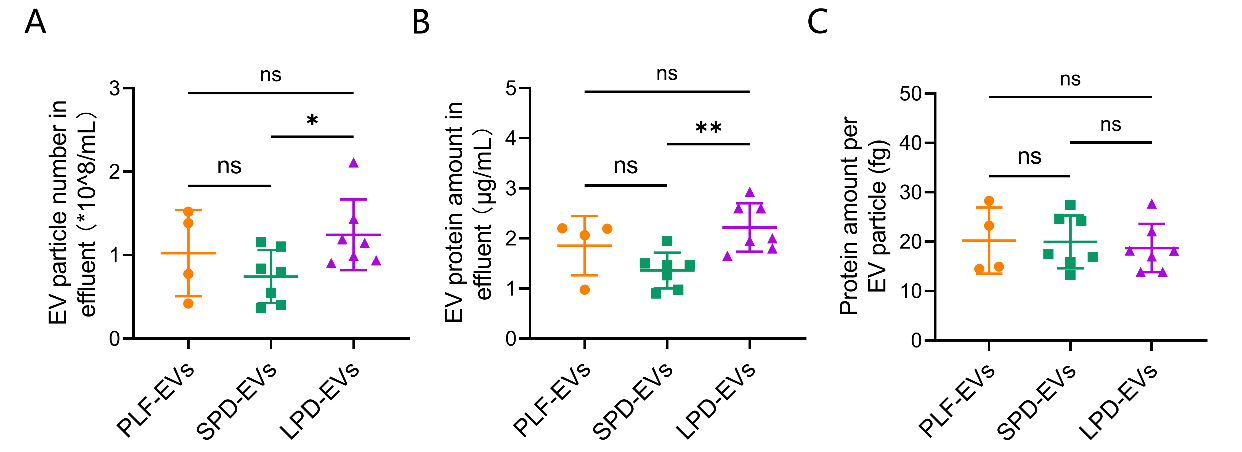


**Fig. S1. Yield of EVs extracted from PLF and PD effluent.** (A) EV particle number of the three groups determined by NTA. (B) EV protein amount of the three groups measured by BCA. (C) The protein amount of single EV particle. ns, not significant, **P* < 0.05, and ***P* < 0.01.


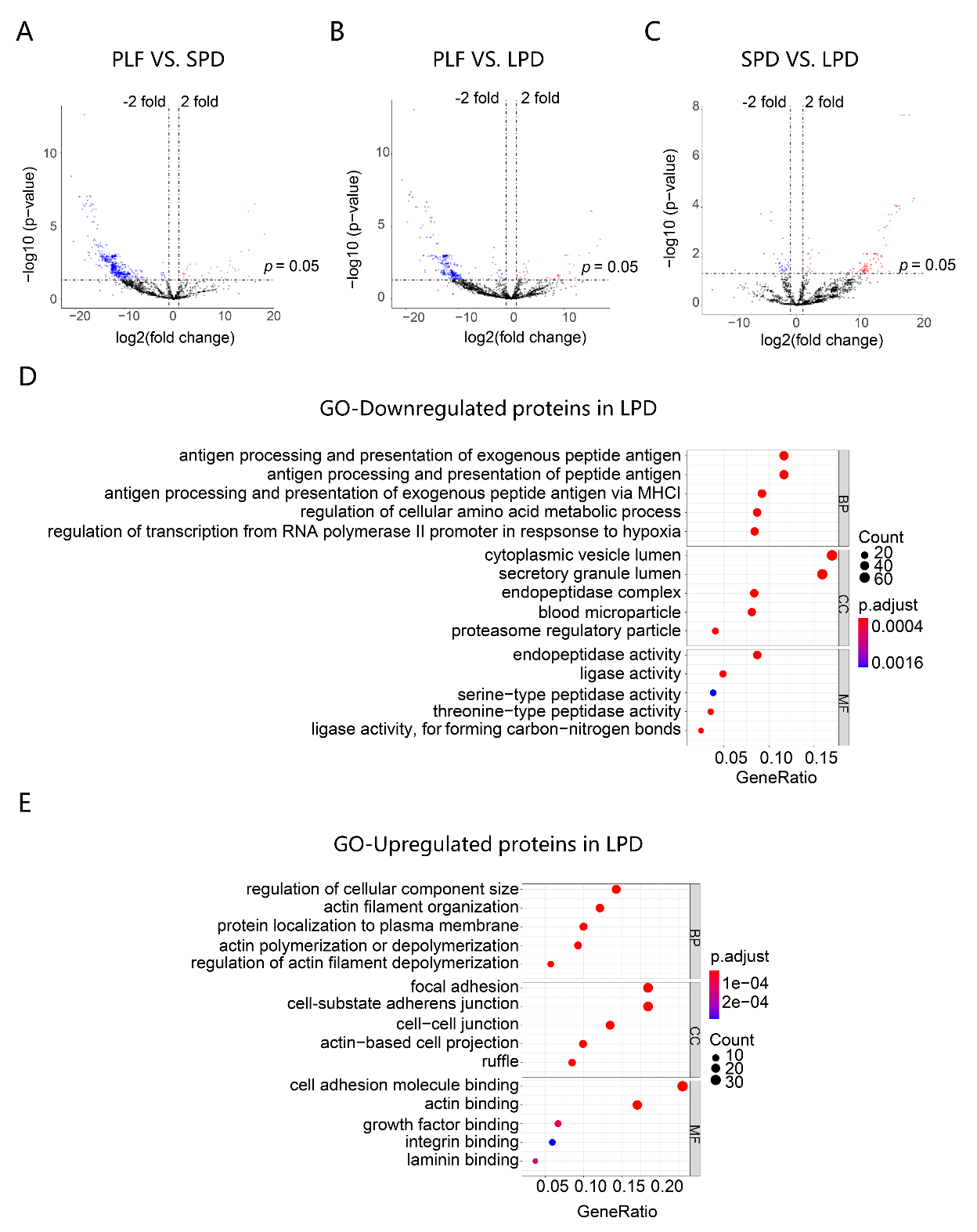


**Fig. S2. Proteomic analyses of the PD effluent-derived EVs.** (A-C) Volcano plot showing a degree of differential expression of EV proteins among PLF, SPD and LPD groups. (A) PLF vs. SPD. (B) PLF vs. LPD. (C) SPD vs. LPD. (D) GO analysis of the down-regulated proteins in LPD-EVs. (E) GO analysis of the up-regulated proteins in LPD-EVs.


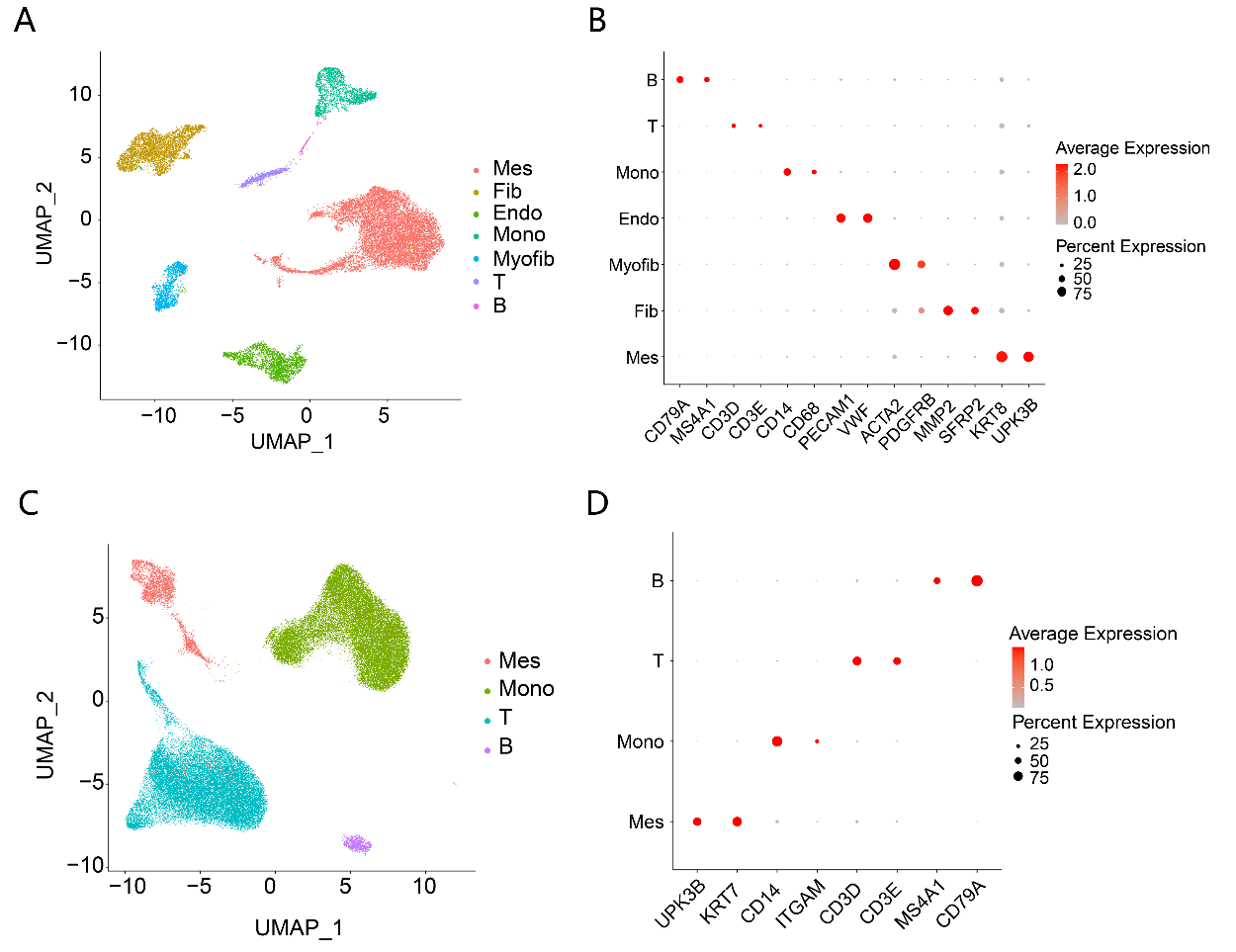


**Fig. S3. Clustering of various cells in normal peritoneal tissues and effluent-derived peritoneal cells from patients undergoing PD.** (A) The Uniform Manifold Approximation and Projection (UMAP) plot showing 7 cell types, normal peritoneal tissues origin. (B) Dot plot showing the expression levels of representative marker genes in each cell type. (C) UMAP plot of 4 cell types identified from effluent-derived peritoneal cells from patients undergoing PD. (D) The expression levels of marker genes in each cell type.


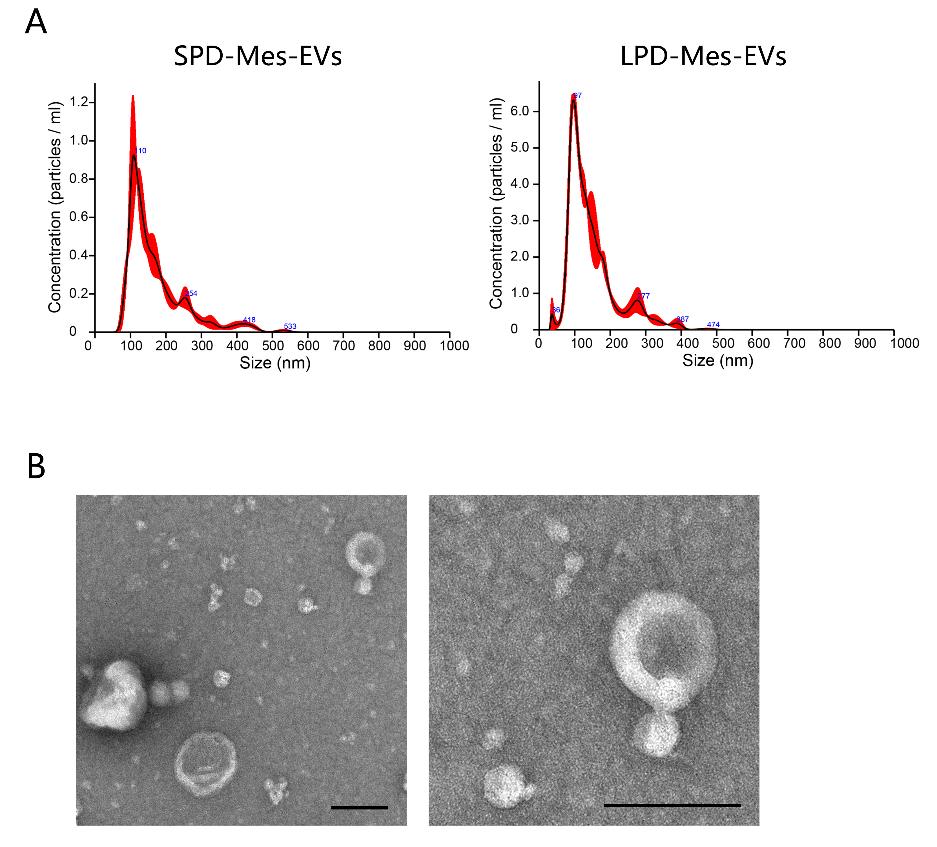


**Fig. S4. Biochemical characterization of EVs isolated from the conditioned media of mesothelial cells.** (A) Representative NTA plot showing the size distribution and concentration of the EVs. (B) Representative electron micrograph of the EVs. Scale bars, 200 nm.


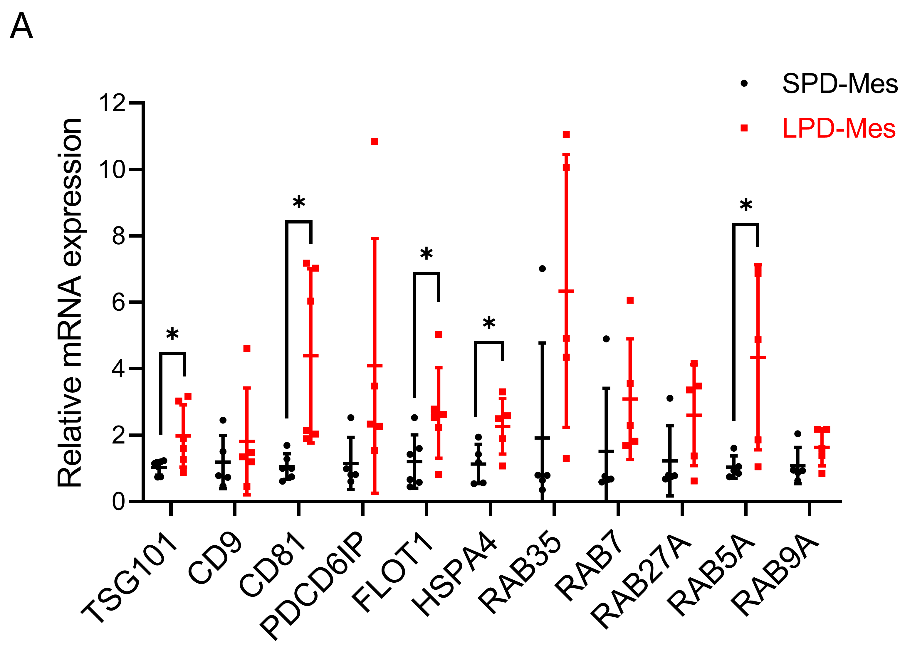


**Fig. S5. The mRNA expression levels of EV biogenesis or release-associated genes upregulated in mesothelial cells from LPD.** (A) Real-time PCR analysis showing relative expression levels of genes (TSG101, CD9, CD81, PDCD6IP, FLOT1, HSPA4, RAB35, RAB7, RAB27A, RAB5A, RAB9A) in mesothelial cells from PD effluent. **P* < 0.05.


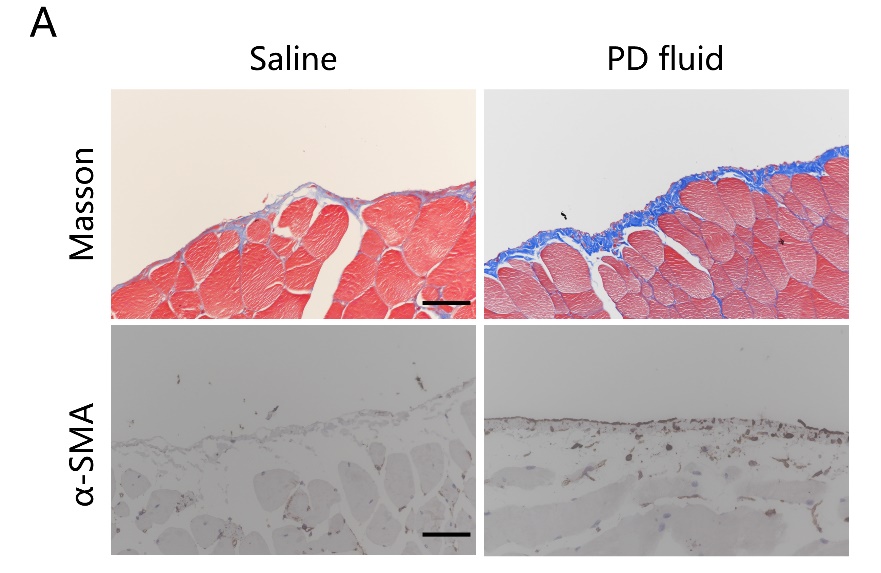


**Fig. S6. The creation of a mouse model of peritoneal fibrosis induced by PD fluid.** (A) Representative masson’s trichrome staining and α-SMA staining of mice peritoneum in saline and PD fluid groups. Scale bars, 50 μm.


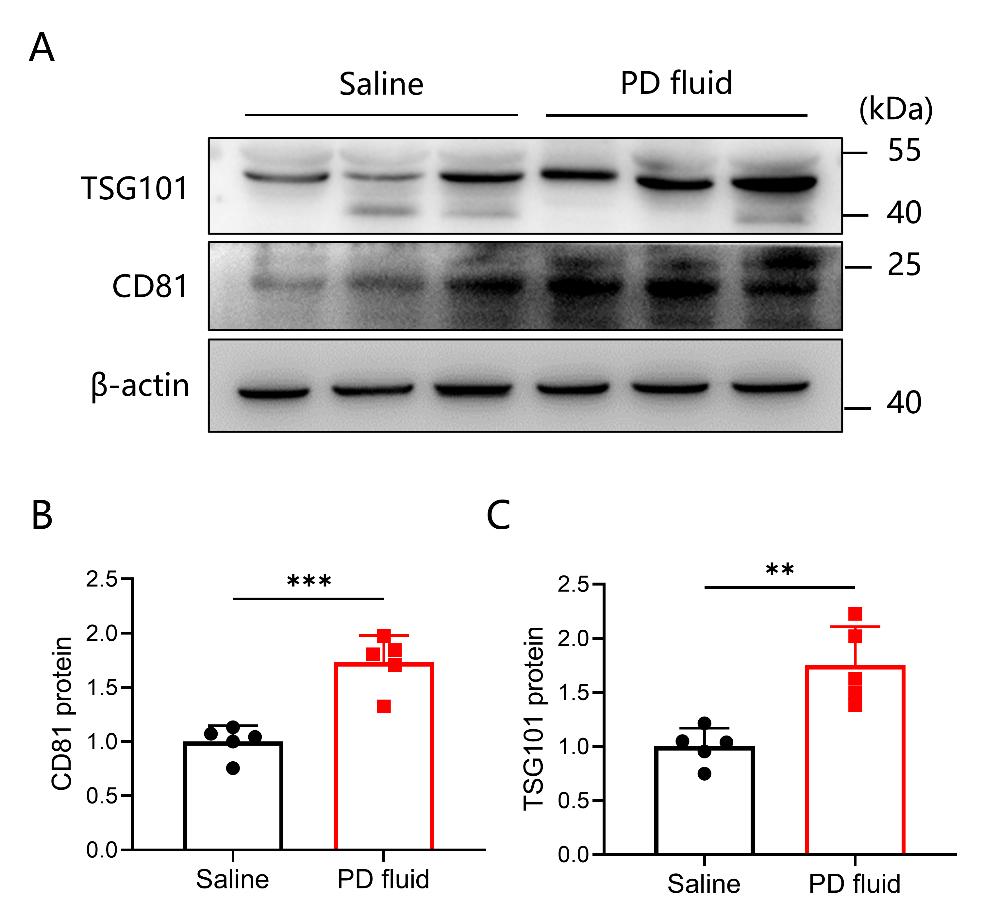


**Fig. S7. The production of EVs increases in a mouse model of peritoneal fibrosis induced by PD fluid.** (A-C) Representative western blot (A) and quantitative (B and C) data of protein expression of CD81 and TGS101 in mice peritoneum. ****P* < 0.001.


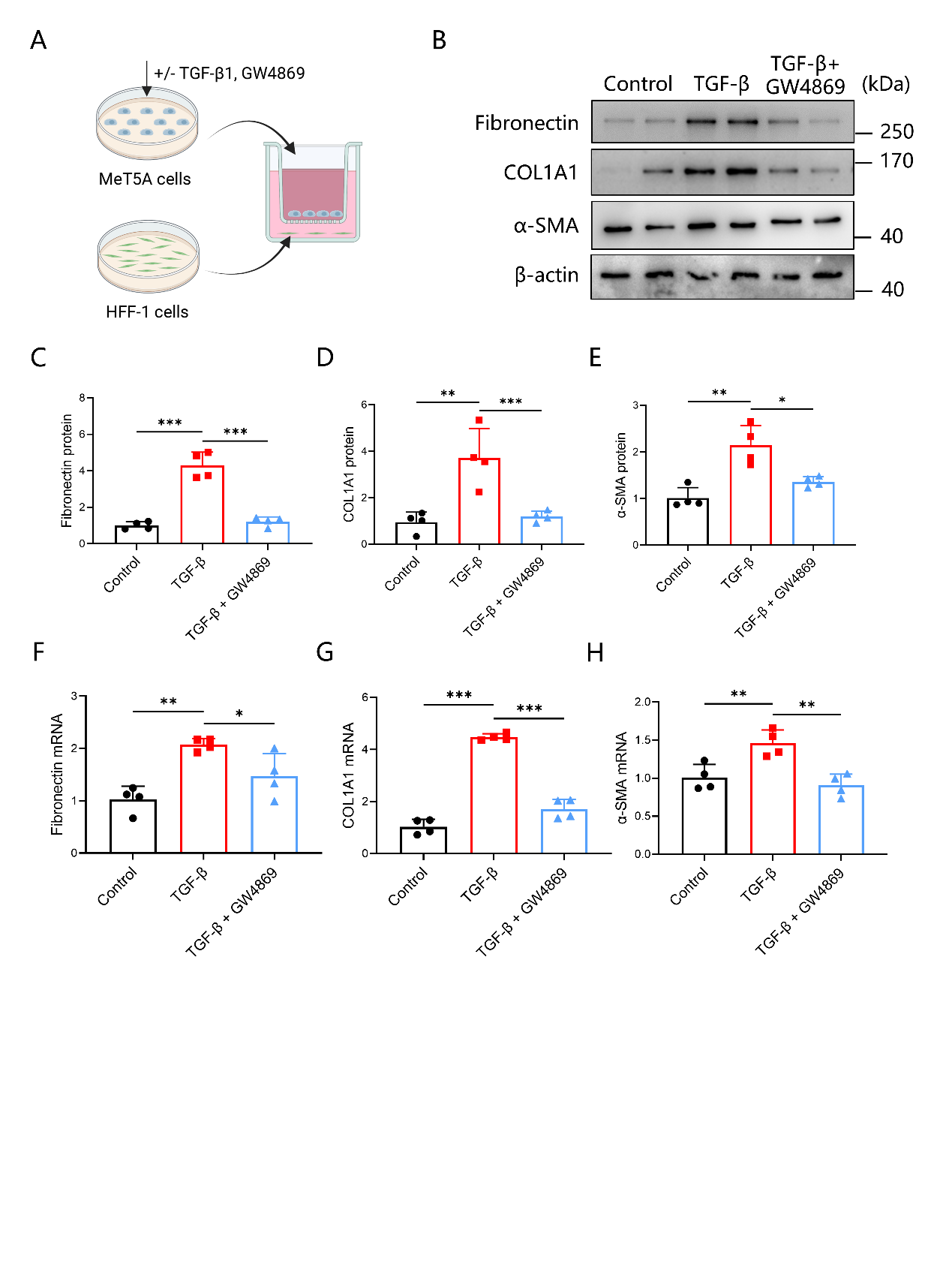


**Fig. S8. Blockade of MeT-5A cell-derived EVs secretion attenuates fibroblast activation.** (A) Schematic diagram of co-culture of MeT5A cells with fibroblasts. (B-H) The mRNA and protein expression levels of fibronectin, COL1A1 and α-SMA in fibroblasts were detected by western blot (B-E) and real-time PCR (F-H) analyses. **P* < 0.05, ***P* < 0.01, and ****P* < 0.001.


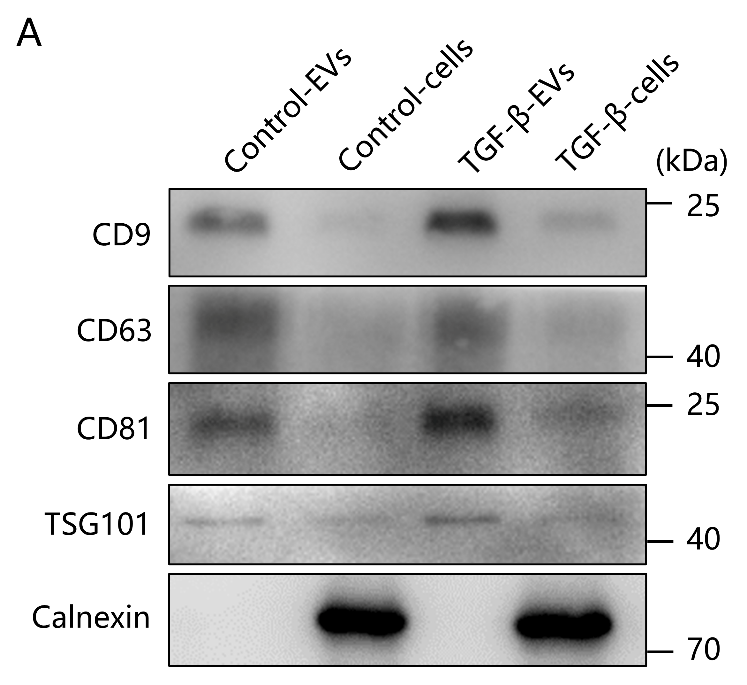


**Fig. S9. EV markers are positive in mesothelial EVs.** (A) western blot analysis of EV positive markers CD9, CD63, CD81 and TSG101 and non-EV marker calnexin.


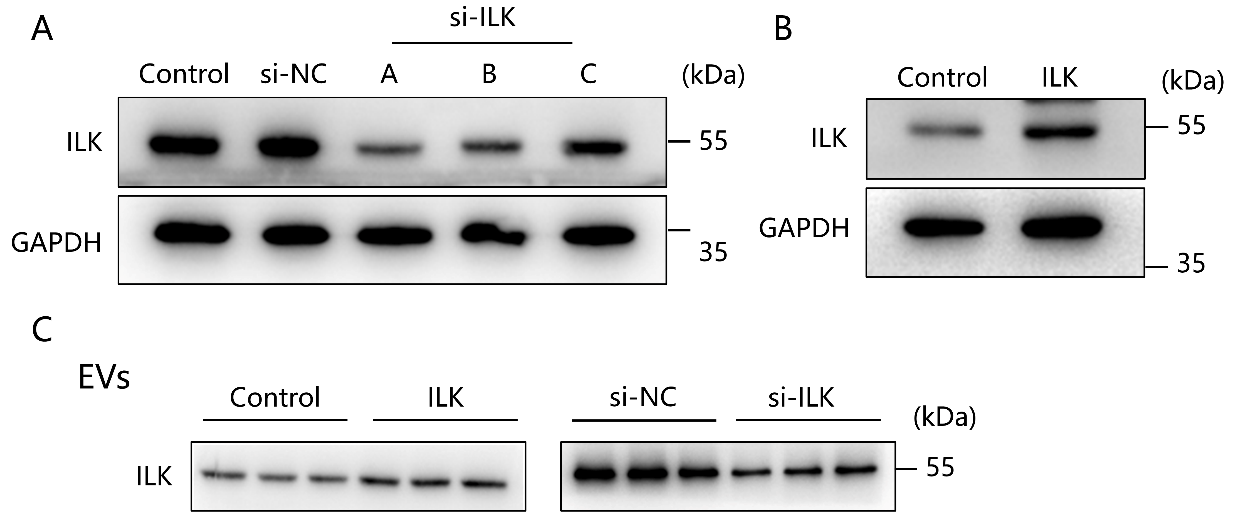


**Fig. S10. The overexpression and knockdown of ILK in mesothelial cells and mesothelial EVs.** (A-B) Western blot analysis showing the effects of ILK knockdown (A) or ILK overexpression (B) on mesothelial cells. (C) EVs were isolated from mesothelial cells transfected with ILK plasmid or si-ILK. Western blot analysis showing the protein expression of ILK in the EVs.
